# Assembly-hub function of ER-localized SNARE proteins in biogenesis of tombusvirus replication compartment

**DOI:** 10.1371/journal.ppat.1007028

**Published:** 2018-05-10

**Authors:** Zsuzsanna Sasvari, Nikolay Kovalev, Paulina Alatriste Gonzalez, Kai Xu, Peter D. Nagy

**Affiliations:** Department of Plant Pathology, University of Kentucky, Lexington, KY, United States of America; Agriculture and Agri-Food Canada, CANADA

## Abstract

Positive-strand RNA viruses assemble numerous membrane-bound viral replicase complexes within large replication compartments to support their replication in infected cells. Yet the detailed mechanism of how given subcellular compartments are subverted by viruses is incompletely understood. Although, Tomato bushy stunt virus (TBSV) uses peroxisomal membranes for replication, in this paper, we show evidence that the ER-resident SNARE (soluble NSF attachment protein receptor) proteins play critical roles in the formation of active replicase complexes in yeast model host and in plants. Depletion of the syntaxin 18-like Ufe1 and Use1, which are components of the ER SNARE complex in the ERAS (ER arrival site) subdomain, in yeast resulted in greatly reduced tombusvirus accumulation. Over-expression of a dominant-negative mutant of either the yeast Ufe1 or the orthologous plant Syp81 syntaxin greatly interferes with tombusvirus replication in yeast and plants, thus further supporting the role of this host protein in tombusvirus replication. Moreover, tombusvirus RNA replication was low in cell-free extracts from yeast with repressed Ufe1 or Use1 expression. We also present evidence for the mislocalization of the tombusviral p33 replication protein to the ER membrane in Ufe1p-depleted yeast cells. The viral p33 replication protein interacts with both Ufe1p and Use1p and co-opts them into the TBSV replication compartment in yeast and plant cells. The co-opted Ufe1 affects the virus-driven membrane contact site formation, sterol-enrichment at replication sites, recruitment of several pro-viral host factors and subversion of the Rab5-positive PE-rich endosomes needed for robust TBSV replication. In summary, we demonstrate a critical role for Ufe1 and Use1 SNARE proteins in TBSV replication and propose that the pro-viral functions of Ufe1 and Use1 are to serve as assembly hubs for the formation of the extensive TBSV replication compartments in cells. Altogether, these findings point clearly at the ERAS subdomain of ER as a critical site for the biogenesis of the TBSV replication compartment.

## Author summary

Viral replication organelles are formed in subcellular compartments during positive-strand RNA virus infections to support robust virus replication. TBSV induces multivesicular body-like structures consisting of aggregated peroxisomes. However, endoplasmic reticulum (ER) and early endosomal proteins and membranes also contribute to the biogenesis of the replication compartment. The authors show that the syntaxin 18-like Ufe1 and Use1 ER SNARE proteins, which are present in ER subdomains called ERAS (ER arrival site), are necessary for the formation of the viral replication organelles. By binding to the p33 replication protein of TBSV, Ufe1 and Use1 serve as an assembly hub for biogenesis of the replication compartment and facilitating the transfer of phospholipids and sterols to the growing sites of viral replication. The advantage of co-opting ER resident SNAREs could be that these proteins constitute very active ER subdomains (ERAS), which might be especially suitable for generation of the extensive membranous viral replication compartment.

## Introduction

Positive-strand (+)RNA viruses assemble numerous membrane-bound viral replicase complexes (VRCs) within large replication compartments/organelles to support their replication in infected cells [1,2,3,4,5]. In addition to the viral-coded replication proteins and the viral RNA(s), the VRCs consist of co-opted host proteins and subcellular membranes. Various (+)RNA viruses subvert or modify different subcellular compartments, such as endoplasmic reticulum (ER), mitochondria, peroxisomes, lysosomes/tonoplasts, chloroplasts or plasma membrane [4,6,7,8]. Yet the detailed mechanism of how given subcellular compartments are subverted by (+)RNA viruses is incompletely understood.

Tomato bushy stunt virus (TBSV) and other tombusviruses code for two viral replication proteins, termed p33 and p92^pol^, which are essential for virus replication [9,10,11]. TBSV forms spherule-like structures (vesicles with narrow openings facing the cytosol) utilizing peroxisomal membranes in plants and yeast, a model host [12,13,14]. TBSV recruits numerous host proteins, whose functions have been studied in some details [15,16,17].

Although TBSV VRCs form on the cytosolic side of the peroxisome boundary membranes at the early stage [13,14,18,19], TBSV replication can efficiently utilize the ER membranes in the absence of peroxisomes [20,21,22]. Accordingly, we have not found essential roles for the best-known peroxisomal biogenesis proteins in TBSV replication [10,23,24,25,26]. In addition, TBSV can utilize the isolated ER membranes efficiently for RNA replication *in vitro*, suggesting that the ER membranes are suitable to assemble the TBSV replicase [27]. Thus, TBSV seems to be able to take advantage of both peroxisome and ER compartments to assemble the VRCs efficiently.

We have previously shown that the yeast Sec39p vesicle-mediated transport protein is needed for TBSV replication [23,28]. Sec39p is part of an ER-localized tethering complex, called Dsl1 complex, that includes Dsl1p and Tip20p. Components of the Dsl1 tethering complex interact with the ER-resident SNARE (soluble NSF attachment protein receptor) proteins Use1p, Ufe1p and Sec20p. The Dsl1 tethering complex in coordination with the SNARE complex forms a special subdomain of ER, called the ER import site (ERIS) or ER arrival site (ERAS), which is involved in the Cop-I vesicle-mediated retrograde transport from the Golgi-to-ER [29,30,31,32,33]. The syntaxin 18-like Ufe1p and Use1p also mediate homotypic, Sey1-independent, ER membrane fusion [34]. However, another important function of the Dsl1 tethering complex and the SNARE complex is to support the ER-dependent peroxisome biogenesis and peroxisome protein distribution [35]. When components of these two complexes are down-regulated or mutated, then the peroxisomal matrix proteins are mislocalized either to the cytosol or the ER. For example, Pex3p peroxisome biogenesis protein is mislocalized to the ER and ER-like tubular structures under these conditions, instead of participating in peroxisome membrane biogenesis [35].

In this paper, we tested if Ufe1p and Use1p SNARE proteins, which are components of the above ERAS/ERIS ER subdomain, affected TBSV replication. We document reduction in TBSV RNA replication in yeast or plants with depleted Ufe1p or Use1p levels. We also present evidence for the mislocalization of the tombusviral p33 replication protein to the ER membrane in Ufe1p-depleted yeast cells. Moreover, over-expression of a dominant-negative mutant of either Ufe1p or the orthologous plant Syp81 greatly interferes with tombusvirus replication in yeast and plants, thus supporting the role of this host protein in tombusvirus replication. The effects of Ufe1p and Use1p seem to be direct based on interaction with the p33 replication protein, co-purification with the tombusvirus replicase, and their presence in the TBSV replication compartment in yeast and plant cells. Altogether, we demonstrate a critical role for Ufe1p and Use1p SNARE proteins in TBSV replication. The many processes affected by the co-opted Ufe1p include the virus-driven membrane contact site formation, sterol-enrichment at replication sites and subversion of the Rab5-positive phosphatidylethanolamine (PE)-rich endosomes needed for robust replication. Based on these findings, we propose that the pro-viral functions of Ufe1p and Use1p are to serve as assembly-hubs for the formation of the extensive TBSV replication compartments in cells. The emerging picture from these findings that the ERAS subdomains of ER are critical sites for the biogenesis of the TBSV replication compartment.

## Results

### The ER-localized syntaxin 18-like Ufe1 and Use1 SNARE proteins interact with the tombusvirus replication protein

**Fig 1:**
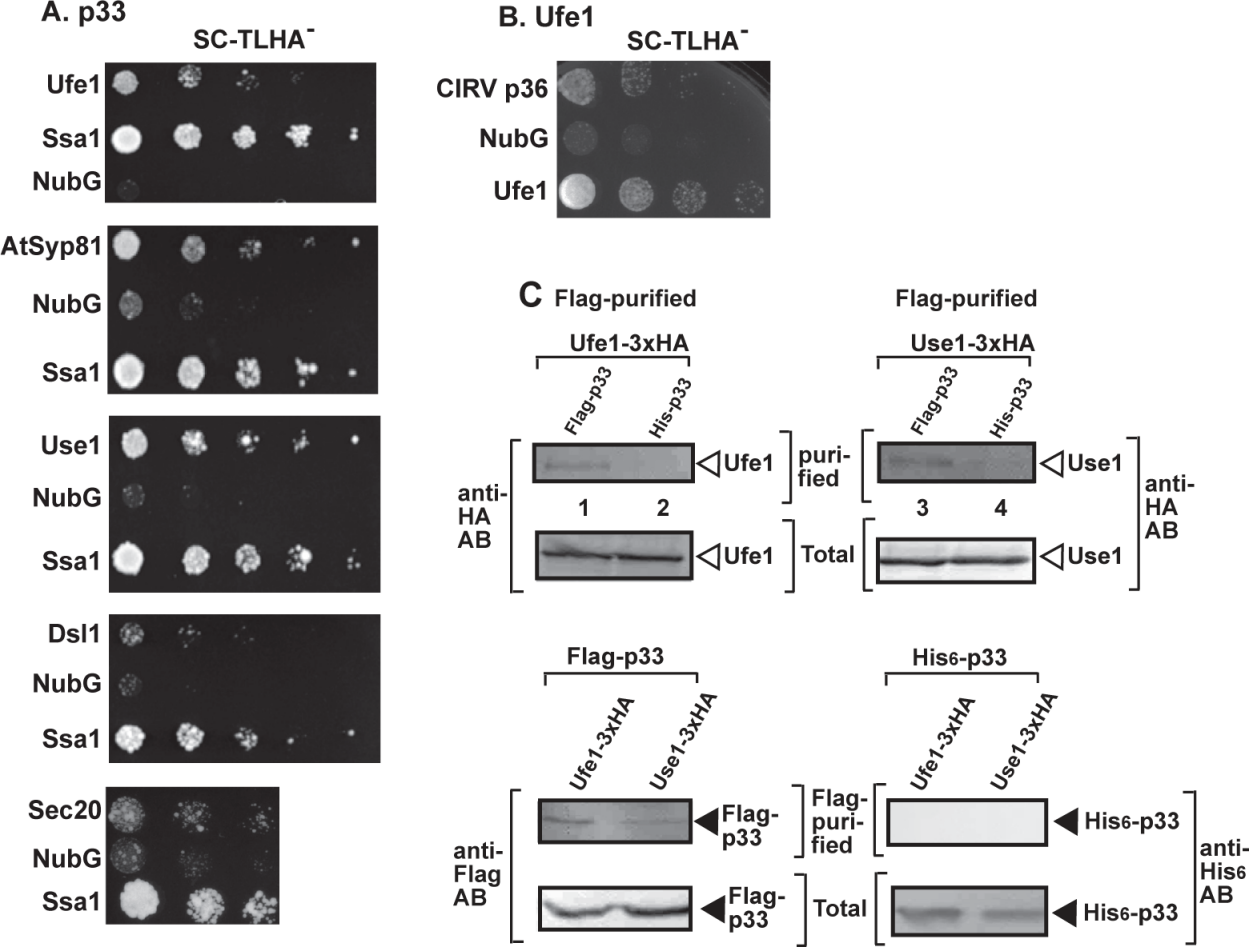
Interaction between TBSV p33 replication protein and the yeast Ufe1p and Use1p SNARE proteins. (A) Top panel: The split ubiquitin assay was used to test binding between the TBSV p33 replication protein and Ufe1p in yeast. The bait p33 was co-expressed with N-terminally-tagged Ufe1p protein. *SSA1* (HSP70 chaperone), and the empty prey vector (NubG) were used as positive and negative controls, respectively. Second-to-fifth panels: The split ubiquitin assay was used to test binding between p33 and AtSyp81 (the plant ortholog of the yeast Ufe1p), Use1p, Dsl1 and Sec20p, respectively, in yeast. Note that Dsl1p protein was C-terminally-tagged. (B) The split ubiquitin assay was used to test binding between the CIRV p36 replication protein and Ufe1p in yeast. See further details in panel A. (C) Co-purification of Ufe1p and Use1p with the p33 replication protein from subcellular membranes. Top panels: Western blot analysis of co-purified HA-tagged cellular Ufe1p protein (lanes 1–2) or Use1p (lanes 3–4) with Flag-affinity purified FLAG/His_6_-p33. Ufe1p and Use1p were detected with anti-HA antibody, while FLAG/His_6_-p33 was detected with anti-FLAG antibody (third and fourth panels). The negative control was His_6_-tagged p33 purified from yeast extracts using a FLAG-affinity column. Second panel: Western blot of total HA-Ufe1p or HA-Use1p in the total yeast extract using anti-HA antibody. Bottom panel: Western blot of total FLAG/His_6_-tagged p33 and His_6_-p33 in the total yeast extract using anti-His antibody.

To explore if cellular components of ER SNAREs (forming the ERAS/ERIS subdomain of ER) and the associated Dsl1 tethering complex interact with the TBSV p33 replication protein, we conducted split-ubiquitin-based membrane yeast two-hybrid assay (MYTH). We found that p33 replication protein interacted with the syntaxin Ufe1p, its plant ortholog AtSyp81, and Use1p, whereas the interaction with either Dsl1p or Sec20p was weak in this assay (Fig 1A). Ufe1p also interacted with the closely related carnation Italian ringspot virus (CIRV) p36 replication protein, which is housed on the outer membranes of mitochondria (Fig 1B).

To further confirm the interactions, we performed co-purification experiments from yeast co-expressing FLAG-tagged p33 and 3xHA-tagged Ufe1p. The haploid yeast strain expressed 3xHA-tagged Ufe1p from the natural promoter and from the yeast chromosome (replacing the untagged wt *UFE1* gene) to support natural level of expression. After detergent-solubilization of the membrane-fraction, the FLAG-p33 was immobilized to the FLAG column. Western blot analysis of the eluted proteins from the column revealed the co-purified Ufe1p (Fig 1C). Similarly prepared yeast extract containing His_6_-p33 was the negative control to exclude non-specific binding by 3xHA-tagged Ufe1p. This co-purification experiment demonstrated the specific binding of Ufe1p to p33 in yeast membranes (Fig 1C, lane 1 versus 2). We obtained similar co-purification result with Use1p SNARE protein (also expressed from the natural promoter and from the yeast chromosome, Fig 1C, lane 3 versus 4), suggesting interaction between the membrane-bound tombusvirus p33 and Use1p.

**Fig 2:**
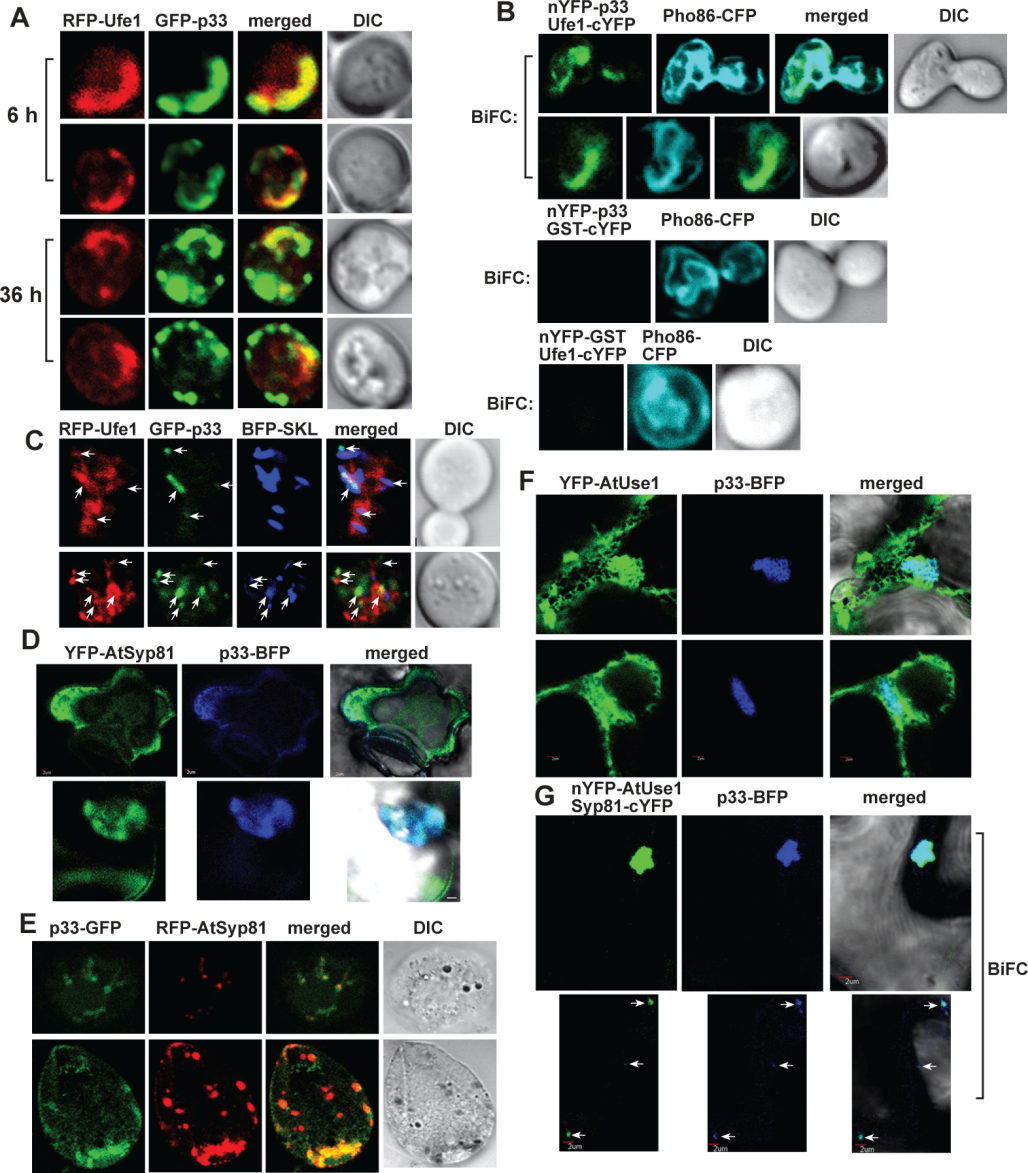
Co-localization of p33 replication protein with Ufe1 and Use1 SNARE proteins. (A) Confocal laser microscopy analysis of subcellular distribution of GFP-tagged p33 and RFP-Ufe1 in wt yeast cells. Images were taken at two time points (as shown). Single frames are presented. (B) Top image: interaction between TBSV nYFP-p33 replication protein and the Ufe1-cYFP protein was detected by BiFC. Co-localization of Pho86-RFP (an ER membrane marker protein) with the BiFC signal (see merged image) demonstrates that the interaction between p33 replication protein and Ufe1 occurs in subdomains of the ER membrane. Control BiFC experiments included GST-cYFP protein in combination with nYFP-p33 replication protein (middle image) and nYFP-GST protein in combination with Ufe1-cYFP (bottom image). (C) Top image: Confocal microscopic images show co-localization of GFP-p33 with both RFP-Ufe1 and BFP-SKL (peroxisomal luminar marker) in the replication compartment. Bottom image shows both proximal localization and co-localization of GFP-p33 and RFP-Ufe1 with BFP-SKL (pointed at by arrows). (D) Confocal microscopic images show co-localization of TBSV p33-BFP replication protein and the YFP-AtSyp81 syntaxin, the ortholog of the yeast Ufe1 protein, *in planta*. Expression of the above proteins from the 35S promoter was done after co-agro-infiltration into *N*. *benthamiana* leaves. Scale bars represent 2 μm. (E) Confocal microscopic images show co-localization of TBSV p33-GFP replication protein and the RFP-AtSyp81 syntaxin in *N*. *benthamiana* protoplasts. (F) Confocal microscopic images show co-localization of TBSV p33-BFP replication protein and the YFP-AtUse1 SNARE protein, the ortholog of the yeast Use1 protein, *in planta*. Expression of the above proteins from the 35S promoter was done after co-agro-infiltration into *N*. *benthamiana* leaves. Scale bars represent 2 μm. (G) Confocal microscopic images show co-localization of TBSV p33-BFP replication protein with AtSyp81 and AtUse1 within the large replication compartment (top images) and smaller replication compartments (depicted with arrows, bottom images) in *N*. *benthamiana* cells. Note that the interaction between of nYFP-AtUse1 and the AtSyp81-cYFP proteins was detected by BiFC. Scale bars represent 2 μm.

Co-expression of the fluorescently tagged p33 and Ufe1p in yeast cells showed the co-localization of a fraction of GFP-p33 and a portion of RFP-Ufe1 at the early time point (6 h after induction of co-expression of these proteins) (Fig 2A), suggesting that p33-Ufe1p interaction might play a role at the early stage of the replication process. Some co-localization of p33 and Ufe1p was observed even at the late time point (36 h) (Fig 2A), which might be due to the continuous expression of these proteins from plasmids in yeast.

To identify the subcellular location of p33-Ufe1p complexes, we performed BiFC experiments in yeast (Fig 2B). These studies indicated that the p33-Ufe1p interaction takes place in dedicated subdomains in the ER membranes (Fig 2B). In addition, co-localization experiments with fluorescently-tagged proteins revealed that p33 is mostly co-localized with BFP-SKL peroxisomal marker and a portion of p33 is co-localized with RFP-Ufe1 (Fig 2C), which were present in the vicinity of the p33-positive peroxisomes. Altogether, these data suggest that p33 interacts with Ufe1p in a special subdomain of ER, which is localized in the vicinity of p33-positive peroxisomes. Ufe1p was not co-localized with BFP-SKL peroxisomal marker in the absence of tombusvirus components, but these proteins were in close proximal locations (S1 Fig). This might be due to the role of the ERAS subdomain, which contains Ufe1p, in the biogenesis of the peroxisomes [35].

Co-expression of p33-BFP and the *Arabidopsis* YFP-Syp81 syntaxin, the ortholog of the yeast Ufe1p [36,37], in *N*. *benthamiana* leaves revealed the co-localization of p33 and AtSyp81 in the large tombusvirus replication compartment (Fig 2D). Similar co-localization of p33-GFP and RFP-Syp81 was observed in *N*. *benthamiana* protoplasts (Fig 2E). Co-expression of p33-BFP and YFP-tagged AtUse1 (the plant ortholog of the yeast Use1p) also showed co-localization of p33 with a portion of AtUse1 (Fig 2F). BiFC-based experiments also revealed that the AtSyp81-AtUse1 complex co-localized with p33 containing replication compartment in *N*. *benthamiana* (Fig 2G). Thus, the co-localization pattern of the plant orthologs Syp81 and Use1 with p33 replication protein in plant cells is comparable to that observed with Ufe1p and Use1p in yeast cells.

### Ufe1 and Use1 SNARE proteins are critical for tombusvirus replication in yeast and in vitro

**Fig 3:**
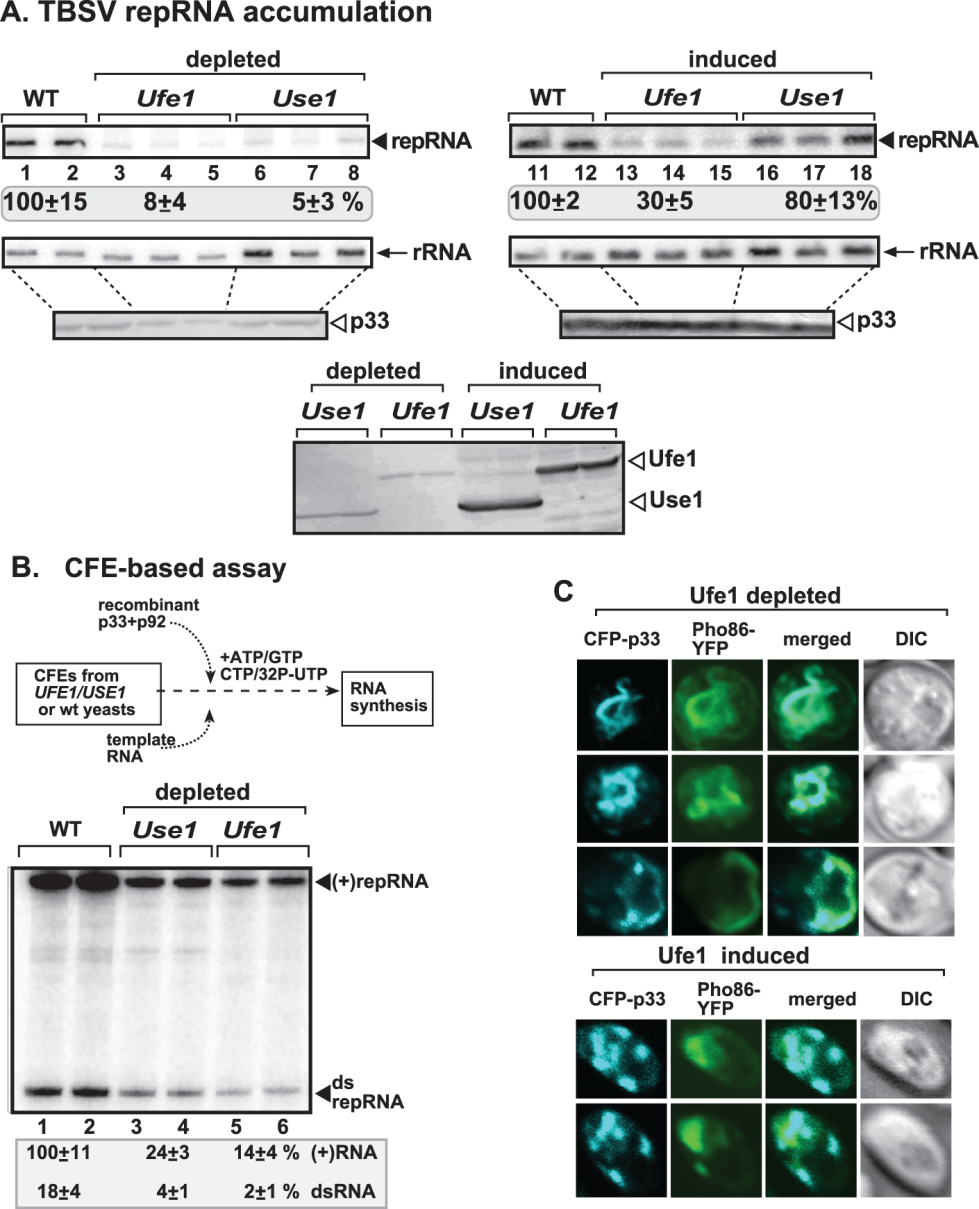
Repression of the expression of either Ufe1p or Use1p SNARE proteins inhibits TBSV repRNA accumulation in yeast. (A) Northern blot analysis of TBSV repRNA using a 3’ end specific probe shows the reduced accumulation of repRNA in GALS::UFE1 or GAL1::USE1 yeast strains grown in glucose media. Viral proteins His_6_-p33 and His_6_-p92 were expressed from plasmids from the copper-inducible *CUP1* promoter, while DI-72(+) repRNA was expressed from the *ADH1* promoter. TBSV replication was induced by growing yeast cells in media supplemented with 50 μM CuSO_4_ at 23°C for 36 hours, whereas the expression of Ufe1p or Use1p was repressed by glucose (lanes 3–8) or induced by galactose (lanes 13–18). Northern blot with 18S ribosomal RNA specific probe was used as a loading control. Bottom images: Western blot analysis of the level of His_6_-p33 with anti-His antibody and Ufe1p or Use1p with anti-HA antibody. (B) Reduced TBSV RNA production by the tombusvirus replicase assembled *in vitro* in cell-free extracts (CFEs) prepared from GALS::UFE1 or GAL1::USE1 yeast strains grown under repressive conditions (in glucose media). Purified recombinant p33 and p92^pol^ replication proteins of TBSV and *in vitro* transcribed TBSV DI-72 (+)repRNA were added to the CFEs prepared from the shown yeast strains. Denaturing PAGE analysis shows the ^32^P-labeled TBSV repRNA products, including the (+)repRNA progeny and the dsRNA intermediate, made by the reconstituted TBSV replicase. Each experiment was repeated. (C) Top images: Confocal laser microscopy analysis of subcellular distribution of CFP-tagged p33 in GALS::UFE1 yeast cells grown under repressive conditions (in glucose media). Note that the co-localization of CFP-p33 with Pho86-RFP (an ER membrane marker protein) demonstrating localization of p33 replication protein in subdomains of the ER membrane. Bottom images: Confocal laser microscopy analysis of subcellular distribution of CFP-tagged p33 in GALS::UFE1 yeast cells grown in galactose media (i.e., inducing condition).

To explore the possible roles of Ufe1p and Use1p in tombusvirus replication, we constructed yeast strains, in which the expression of either Ufe1p or Use1p could be regulated due to the replacement of the native promoters with *GAL1* promoter in the haploid yeast chromosomes (GALS::UFE1 and GAL1::USE1 yeast strains). Repression of Ufe1p expression on glucose media resulted in a low level TBSV repRNA replication (8% of that seen in wt yeast, compare lanes 3–5 to 1–2, Fig 3A). Repression of Use1p expression also reduced TBSV repRNA accumulation below 10% level (Fig 3A). Western blot analysis revealed that the tombusvirus replication proteins were expressed, while the Ufe1p and Use1p levels were low, but still detectable, likely due to the stability of these proteins in yeast. Separate induction of Ufe1p and Use1p increased TBSV repRNA accumulation to 30% and 80%, respectively, of the level supported in wt yeast (Fig 3A), indicating that the defect in the Ufe1p or Use1p depleted strains can be partially complemented.

To test if Ufe1p plays a role in tombusvirus replicase assembly, we reconstituted the tombusvirus replicase *in vitro* by using (+)repRNA transcripts and purified recombinant p33 and p92^pol^ replication proteins in cell-free extracts (CFE) prepared from either wt yeast or yeast with depleted Ufe1p level (Fig 3B). Nondenaturing PAGE analysis of the *in vitro* replicase products revealed ~9-fold reduction in dsRNA replication intermediate and ~7-fold reduction for (+)ssRNA products in CFE with depleted Ufe1p in comparison with the RNA replication supported by WT CFE (Fig 3B, lanes 5–6 versus 1–2). The observation that both the new (-)RNA (present in dsRNA) and (+)RNA products were decreased when CFE contained depleted Ufe1p level suggests that Ufe1p likely plays a role during the replicase assembly steps, prior to (-)RNA and (+)RNA synthesis steps. Interestingly, the reconstituted replicase in CFE containing depleted Use1p level also supported low level of (-)RNA or (+)RNA synthesis (~5-fold reduction, Fig 3B, lanes 3–4). Thus, both Use1p and Ufe1p likely facilitate replicase assembly, but their roles are not redundant.

Confocal microscopic analysis of yeast depleted in Ufe1p revealed ER localization of p33 replication protein (Fig 3C, top panels), instead of the canonical peroxisomal localization [21,38]. Moreover, p33 distribution was somewhat unusual, since in addition to small punctate structures, large portion of the ER membranes contained p33, suggesting the inhibition of discrete replication compartment formation, which is a characteristic feature in wt yeast cells. In the Ufe1p complemented yeast, p33 distribution mostly showed the characteristic large punctate structures (Fig 3C, bottom panels), suggesting the formation of normal viral replication compartments. However, a portion of p33 molecules was still co-localized with the ER marker in the Ufe1p complemented yeast (Fig 3C, bottom panels), which only partially complemented the replication defect of TBSV in this yeast (Fig 3A). These data are in agreement with a model that the ER-resident Ufe1p SNARE protein plays a significant role in the formation of the tombusvirus replication compartment.

**Fig 4:**
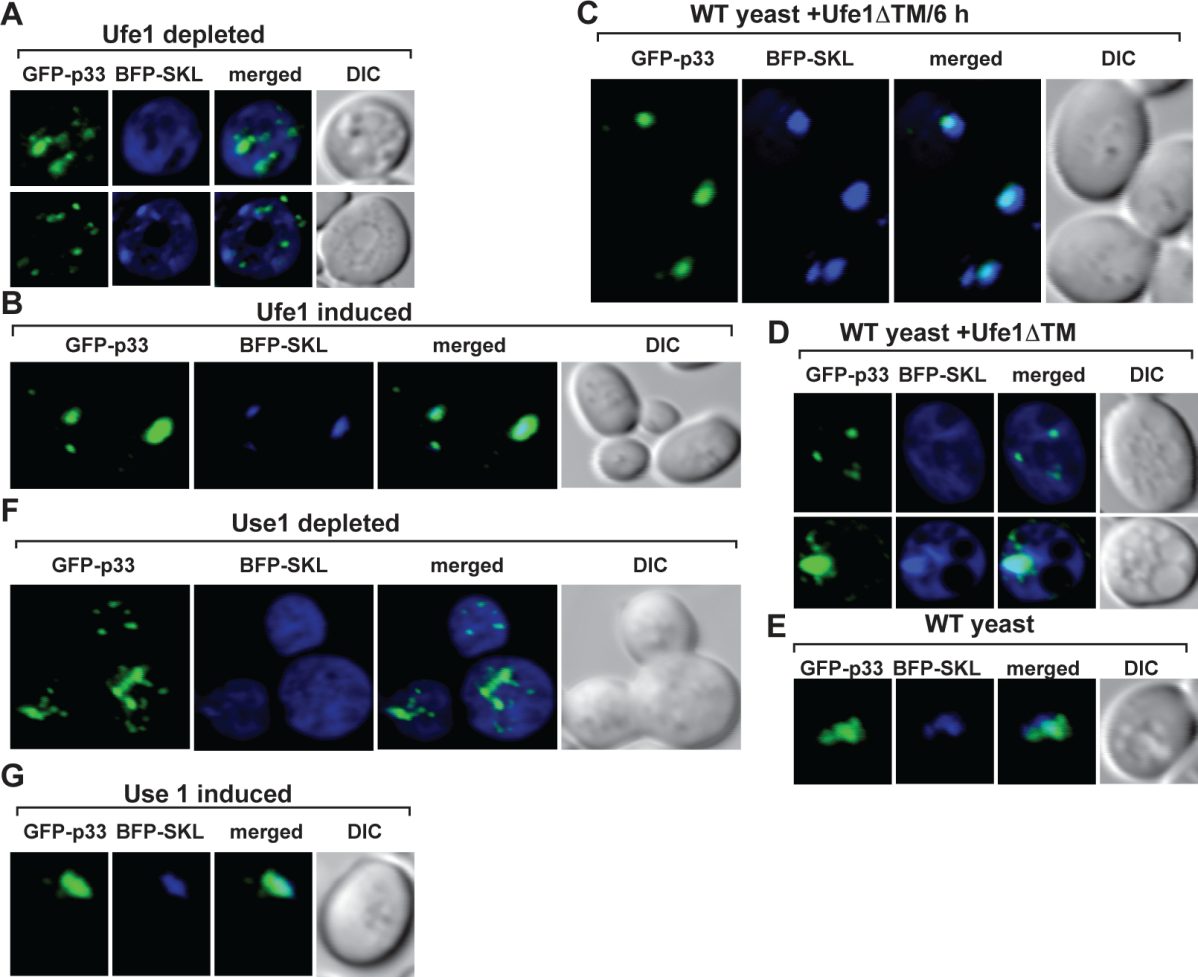
Repression of the expression of either Ufe1p or Use1p SNARE proteins inhibits peroxisome biogenesis in yeast. (A) Confocal laser microscopy analysis of subcellular distribution of BFP-SKL peroxisomal luminar marker in GALS::UFE1 yeast cells grown under repressive conditions (in glucose media). Note that the cytosolic distribution of BFP-SKL and the lack of co-localization of with GFP-p33 demonstrating inhibition of peroxisome biogenesis. (B) Confocal laser microscopy analysis of subcellular distribution of BFP-SKL in GALS::UFE1 yeast cells grown in galactose media (i.e., inducing condition). (C-D) The effect of expression of the dominant-negative Ufe1p mutant (Ufe1ΔTM) on subcellular distribution of BFP-SKL in wt yeast strain. RFP-Ufe1ΔTM was expressed 6 hours after induction of BFP-SKL and GFP-p33 (panel C) or simultaneous expression of the three proteins from plasmids (panel D). (E) WT yeast control not expressing Ufe1ΔTM. (F) Confocal laser microscopy analysis of subcellular distribution of BFP-SKL peroxisomal luminar marker in GAL1::USE1 yeast cells grown under repressive conditions (in glucose media). Note that the cytosolic distribution of BFP-SKL and the lack of co-localization of with GFP-p33 demonstrating inhibition of peroxisome biogenesis. (G) Confocal laser microscopy analysis of subcellular distribution of BFP-SKL in GAL1::USE1 yeast cells grown under inducing conditions.

The main subverted organellar component of the TBSV replication compartment is the peroxisome [13,14,18,38], therefore we tested if peroxisome formation/maintenance was affected by depletion of Ufe1p in yeast. BFP-SKL, a peroxisomal luminar marker, is co-localized with the p33-induced viral replication compartment when expression of Ufe1p is induced (Fig 4B). However, depletion of Ufe1p in yeast resulted in cytosolic distribution of BFP-SKL and different localization pattern from GFP-p33 (Fig 4A), confirming the absence of peroxisomes. Similarly, depletion of Use1p SNARE protein also led to cytosolic distribution of BFP-SKL (Fig 4F), in contrast with co-localization of BFP-SKL with the p33 within the large viral replication compartment when expression of Use1p is induced (Fig 4G). Therefore, Ufe1p and Use1p are required for formation of TBSV replication compartments and biogenesis of peroxisomes.

**Fig 5:**
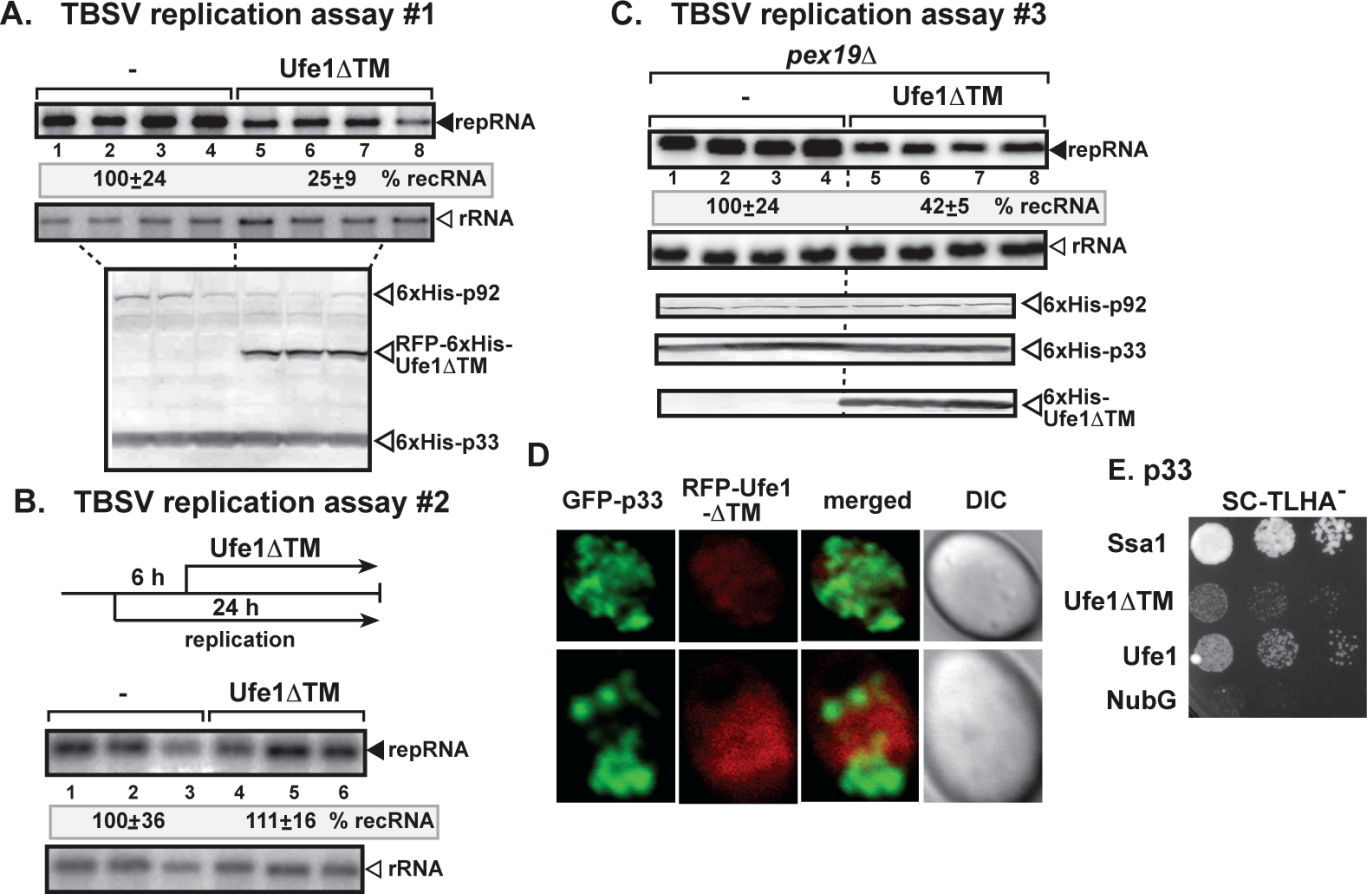
Expression of dominant-negative mutant of Ufe1p inhibits TBSV repRNA accumulation in yeast. (A) Northern blot analysis of TBSV repRNA shows the reduced accumulation of repRNA in wt yeast strain expressing RFP-Ufe1ΔTM. Viral proteins His_6_-p33 and His_6_-p92 were expressed from plasmids from the copper-inducible *CUP1* promoter, while DI-72(+) repRNA was expressed from the *ADH1* promoter. TBSV replication was induced by growing yeast cells in media supplemented with 50 μM CuSO_4_ at 23°C for 36 hours, whereas the co-expression of RFP-Ufe1ΔTM was induced by galactose (lanes 5–8). Northern blot with 18S ribosomal RNA specific probe was used as a loading control. Bottom image: Western blot analysis of the expression levels of His_6_-p33, His_6_-p92 and RFP-Ufe1ΔTM with anti-His antibody. (B) Northern blot analysis of TBSV repRNA shows the lack of inhibition of repRNA accumulation in wt yeast strain when RFP-Ufe1ΔTM was expressed 6 hours after launching TBSV repRNA replication. See further details in panel A. Each experiment was repeated. (C) Northern blot analysis of TBSV repRNA shows the reduced accumulation of repRNA in *pex19Δ* yeast strain expressing RFP-Ufe1ΔTM. See further details in panel A. Each experiment was repeated. (D) Confocal laser microscopy analysis of subcellular distribution of GFP-tagged p33 and RFP-Ufe1ΔTM in wt yeast cells. Note that the different pattern of localization of GFP-tagged p33 and RFP-Ufe1ΔTM, which is mostly cytosolic due to the deletion of the transmembrane region. (E) The split ubiquitin assay was used to test binding between the TBSV p33 replication protein and Ufe1ΔTM in yeast. The bait p33 was co-expressed with N-terminally-tagged Ufe1ΔTM protein. *SSA1* (HSP70 chaperone), and the empty prey vector (NubG) were used as positive and negative controls, respectively.

Since we could not completely deplete the essential Ufe1p in GALS::UFE1 yeast strain (Fig 3A), we also tested the effect of expression of the dominant negative mutant of Ufe1p (ΔTM, missing the transmembrane domain) [34] in wt yeast. Ufe1ΔTM inhibited TBSV repRNA accumulation by ~4-fold when co-expressed with the tombusvirus replication proteins (Fig 5A). In contrast, delayed expression of Ufe1ΔTM had no inhibitory effect on virus replication (Fig 5B), suggesting that the pro-viral function of Ufe1p is likely performed at the early time point of replication. Expression of Ufe1ΔTM inhibited the biogenesis of peroxisomes in WT yeast as indicated by the mostly cytosolic distribution of BFP-SKL peroxisomal luminar marker (Fig 4D). Unlike in WT yeast with strong co-localization, BFP-SKL was poorly co-localized with GFP-tagged p33 in the presence of Ufe1ΔTM (Fig 4D and 4E). Delayed expression of Ufe1ΔTM had no inhibitory effect on the efficient co-localization of BFP-SKL and GFP-p33 (Fig 4C), further supporting that the pro-viral role of Ufe1p is at the early steps in TBSV replication.

Expression of Ufe1ΔTM also inhibited TBSV repRNA replication by ~2.5-fold in the absence of peroxisomes (due to deletion of the *PEX19* peroxisome biogenesis gene in yeast) (Fig 5C), when TBSV replication is carried out in the ER membranes [18,21]. The localization of Ufe1ΔTM was diffused, most likely in the cytosol, and did not overlap with GFP-p33 (Fig 5D). However, we did detect weak interaction between p33 and Ufe1ΔTM in the MYTH assay (Fig 5E). Thus, it seems that only a fraction of Ufe1ΔTM is involved in inhibiting TBSV replication through dominant negative effect on the wt Ufe1p [34] or inhibition of p33 function due to nonproductive interaction between p33 and Ufe1ΔTM. These data support the theme that the ERAS subdomain of ER containing Ufe1p and Use1p SNAREs is critical for tombusvirus replication, but not the peroxisomal membranes per se.

The central role of the ERAS subdomain of ER in tombusvirus replication is further supported by depletion of Sec20p SNARE protein, which forms a complex with Ufe1p and Use1p SNARE proteins in the ER (shown schematically in S2A Fig). Down-regulation of Sec20p has led to a ~3-fold decrease in TBSV repRNA accumulation in yeast (S2B Fig). A comparable level of reduction in *in vitro* replication of TBSV repRNA was observed in CFE prepared from Sec20p-depleted yeast, in which the tombusvirus replicase was reconstituted *in vitro* by using purified recombinant p33 and p92 replication proteins (S2C Fig). Similarly, expression of Dsl1 mutants in yeast inhibited TBSV repRNA accumulation by ~90% (S2D Fig) and interfered with the *in vitro* assembly/activity of the TBSV replicase in CFE prepared from yeast expressing dsl1 mutant protein (S2E Fig). Expression of a Tip20p mutant, which is the third component of the Dsl1-tethering complex in ERAS (S2A Fig), inhibited TBSV replication by ~5-fold (S2F Fig). Altogether, these studies have established that Ufe1p and components of the ERAS/ERIS subdomain of ER, including SNAREs and the Dsl1 tethering complex, are critical for robust and efficient TBSV replication in yeast.

In addition to Ufe1p, which can shape ER morphology (tubules or vesicular sheets) via supporting homotypic ER membrane fusion, the atlastin-like Sey1p (dynamin-like GTPase) is also involved in similar homotypic ER membrane fusion [39,40,41]. Therefore, we tested if deletion of *SEY1* could affect TBSV replication in yeast. These experiments revealed that deletion of *SEY1* or double-deletion of *SEY1* and *YOP1* reticulon (which stabilizes ER tubules via inducing membrane curvature) did not affect TBSV replication in yeast (S3A and S3B Fig). Also, the ER-resident RFP-Sey1p did not co-localize with GFP-p33 (S3C Fig), suggesting that these proteins are present in different subcellular locations. Thus, based on the *SEY1* and *YOP1* data above, we suggest that the homotypic ER membrane fusion function of Ufe1p is unlikely to affect TBSV replication indirectly through regulating ER morphology.

### The Ufe1 ortholog SYP81 syntaxin is required for tombusvirus replication in plants

**Fig 6:**
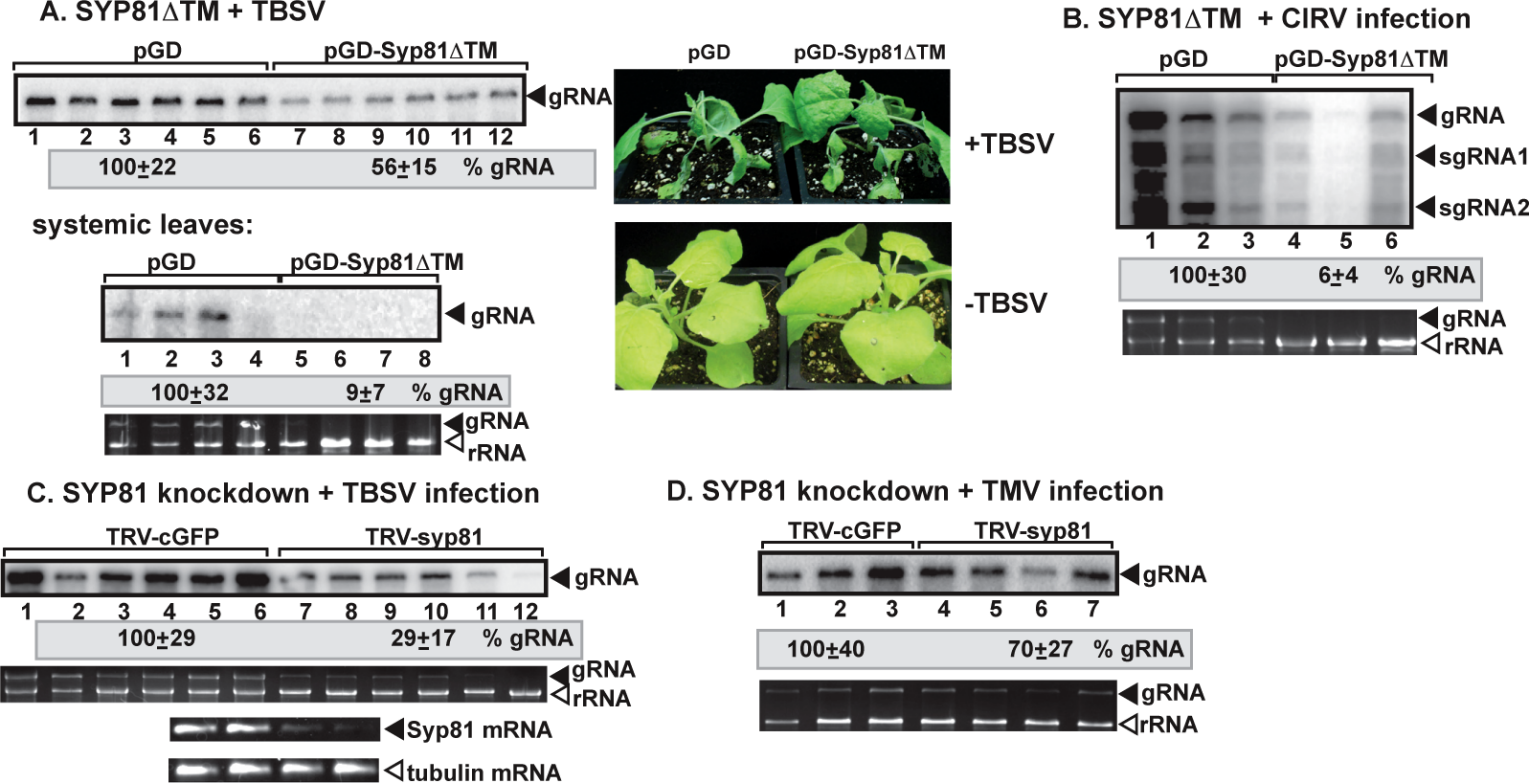
Knockdown of *SYP81* gene inhibits TBSV RNA replication in *N*. *benthamiana* plants. (A) Top panel: Reduced accumulation of TBSV RNA in *N*. *benthamiana* leaves expressing Syp81ΔTM, the dominant-negative mutant of Syp81 syntaxin, the ortholog of the yeast Ufe1 protein. Expression of Syp81ΔTM from the 35S promoter was done after agro-infiltration into *N*. *benthamiana* leaves. Second panel: Accumulation of the TBSV genomic (g)RNA in the systemically-infected *N*. *benthamiana* plants transiently expressing Syp81ΔTM. (B) Top panel: Reduced accumulation of CIRV RNAs in *N*. *benthamiana* leaves expressing Syp81ΔTM. Expression of Syp81ΔTM from the 35S promoter was done after agro-infiltration into *N*. *benthamiana* leaves. (C) Accumulation of the TBSV genomic (g)RNA in Syp81 knockdown *N*. *benthamiana* plants 3 days post-inoculation, based on Northern blot analysis. Inoculation with TBSV gRNA was done 9 days after silencing of Syp81 expression by sap inoculation. VIGS was performed via agroinfiltration of tobacco rattle virus (TRV) vectors carrying NtSyp81 sequence or the TRV-cGFP vector (as a control). Second panel: Ribosomal RNA is shown as a loading control. Note that the TBSV genomic RNA is also visible in the gel. Third panel: RT-PCR analysis of NbSyp81 mRNA level in the silenced and control plants. Fourth panel: RT-PCR analysis of *TUBULIN* mRNA level in the silenced and control plants. Each experiment was repeated. (D) Accumulation of the unrelated TMV genomic RNA in Syp81 knockdown *N*. *benthamiana* plants 3 days post-inoculation, based on Northern blot analysis. See further details in panel C.

To explore if the *Arabidopsis* ER-resident SYP81 SNARE, which is orthologous with the yeast Ufe1p and the mammalian syntaxin 18 [42], is needed for tombusvirus replication, we transiently expressed dominant negative SYP81 (SYP81-ΔTM, missing the trans-membrane region) in *Nicotiana benthamiana* to inhibit the activity of wt SYP81. Then, the same leaves were inoculated with either TBSV or the closely related mitochondrial CIRV. Northern blot analysis revealed ~2-fold reduction in TBSV genomic RNA accumulation in the inoculated leaves and ~10-fold reduction in the systemically-infected leaves (Fig 6A). CIRV gRNA accumulation was reduced by ~10-fold by the expression of SYP81-ΔTM in *N*. *benthamiana* (Fig 6B).

We also used a virus-induced gene silencing (VIGS) approach to deplete SYP81 level in *N*. *benthamiana* that resulted in down-regulation of *SYP81* mRNA (Fig 6C). Replication of TBSV gRNA was decreased by ~4-fold in the *SYP81* knockdown plants when compared to the nonsilenced plants (Fig 6C, lanes 7–12 versus 1–6). On the contrary, the accumulation of the unrelated *Tobacco mosaic virus*, also a (+)RNA virus, was decreased only by 30% in *SYP81* knockdown plants (Fig 6D). Altogether, these data suggest that SYP81 is specifically required for tombusvirus (TBSV and CIRV) accumulation in plants. Silencing of *SYP81* also inhibited the growth of the newly emerging leaves, which stayed much smaller than in nonsilenced plants and the apical terminal shoots stopped growing. In addition, silencing of the plant *DSL1* by VIGS also led to robust inhibition of TBSV replication in *N*. *benthamiana* (S4 Fig). Thus, similar to the results obtained in yeast, the ERAS subdomain of ER, including the SNARE complex and the Dsl1 tethering complex, is a major factor in tombusvirus replication in plants.

### Ufe1 and Use1 SNAREs are required for the formation of virus-induced membrane contact sites needed for tombusvirus replication in yeast

To gain insights into the pro-viral function of Ufe1p, we tested if TBSV could efficiently co-opt other pro-viral cellular proteins when yeast co-expressed the dominant-negative Ufe1ΔTM. We purified FLAG-p33 replication protein from detergent-solubilized membrane fractions from wt yeast and yeast co-expressing Ufe1ΔTM, followed by Western blotting to measure the co-purified cellular proteins.

Western blot analysis of set of co-purified pro-viral host factors revealed that several additional co-opted cellular factors were present in reduced (by 35-to-55%) amounts in the viral replicase obtained from yeast expressing Ufe1ΔTM versus WT yeast control (Table 1). These included Pex19p shuttle protein, which is subverted by p33/p92 to target the viral proteins to peroxisomal membranes [18], Vps4p AAA+ ATPase (an ESCRT protein), required for VRC assembly, and co-factors involved in viral RNA synthesis, such as eEF1A translation elongation factor, eIF4AIII-like RH2 DEAD-box helicase and Tdh2p (GAPDH), all of which were recruited into the viral replicase with reduced efficiency in yeast expressing Ufe1ΔTM. (Table 1). In addition, we found 30-to-40% less amount of co-purified Osh6p and Vap27-1 from yeast co-expressing Ufe1ΔTM than from wt yeast (Table 1). Both VAP27-1 (plant ortholog of the yeast Scs2 VAP protein) and Osh6p are required for p33-induced sterol enrichment within the replication compartment [43]. The reduced recruitment of these pro-viral cellular proteins into the viral replication compartment in the presence of Ufe1ΔTM might explain, at least to some degree, the inhibitory effect of Ufe1ΔTM on TBSV replication in yeast.

**Table 1:** Co-purified host proteins with p33 from yeast expressing Ufe1ΔTM.

Protein	Pro-viral function	percentage
**Ded1 helicase**	**(+)-strand synthesis**	**98±10**
**Cdc34 E2 enzyme, ub**	**Ubiquitination of p33**	**109±14**
**Osh6 ORP (OSBP1-like)**	**oxysterol binding**	**59±24**
**Pbp2 RNA binding**	**Not known**	**122±15**
**Rpn11 deubiquitinase**	**Recruitment of Ded1**	**103±6**
**Pex19**	**Targeting of p33 to peroxisomes**	**56±13**
**RH2 DEAD-box helicase**	**(+)-strand synthesis**	**70±18**
**Tdh2 GAPDH**	**(+)-strand synthesis**	**64±17**
**Tef1 eEF1A**	**VRC assembly, (-)-strand synthesis, p33 stability**	**67±25**
**Vap27-1 (Scs2-like)**	**MCS formation, sterol**	**68±30**
**Vps4 AAA ATPase**	**VRC assembly**	**44±27**
**Vps23 ESCRT I**	**VRC assembly**	**106±7**

The amount of a given host protein co-purified with p33 from WT yeast (lacking Ufe1ΔTM) is taken as 100%. Each purification was repeated at least 3 times.

On the other hand, several co-opted cellular host factors, such as DDX3-like Ded1p DEAD-box helicase, Cdc34 ubiquitin-conjugating enzyme, Rpn11 deubiqutinase and Vps23p ESCRT factor were co-purified with p33 replication protein as efficiently from yeast expressing Ufe1ΔTM as from wt yeast (Table 1). Because the recruitment of not all the cellular proteins are affected by Ufe1ΔTM expression (Table 1), it seems that Ufe1p is specifically involved in the pre-assembly of a selected group of co-opted host proteins and the replication proteins prior to the final assembly of VRCs.

**Fig 7:**
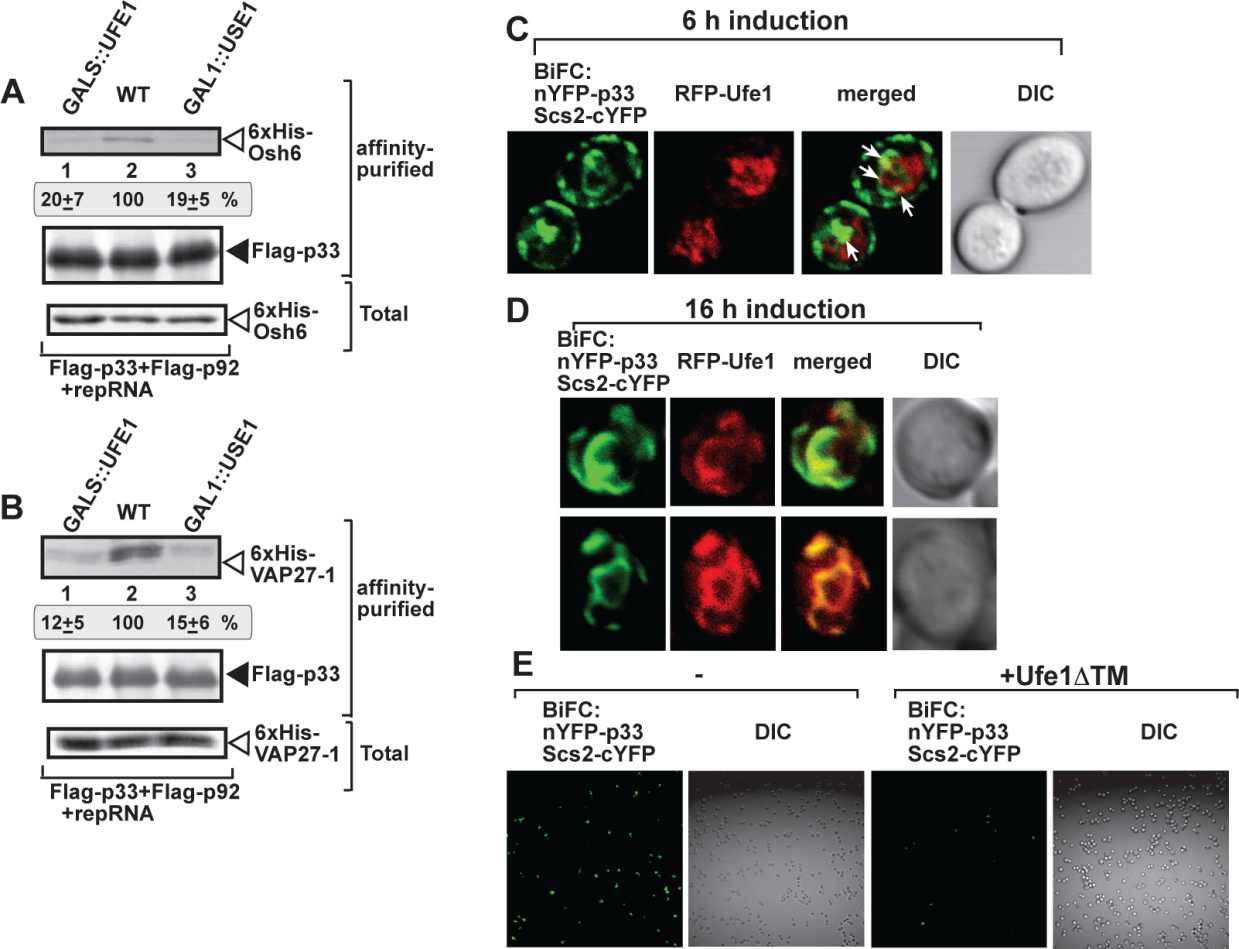
P33 replication protein interacts with Scs2p VAP protein in the vicinity of Ufe1-positive membranes. (A) Co-purification of 6xHis-tagged Osh6 with the p33 and p92 replication proteins from subcellular membranes from yeasts with depleted Ufe1p (GALS::UFE1), depleted Use1p (GAL1::USE1) or WT yeasts. Top panel: Western blot analysis of co-purified His_6_-Osh6p protein with Flag-affinity purified FLAG-p33. His_6_-Osh6p was detected with anti-6xHis antibody, while FLAG-p33 was detected with anti-FLAG antibody (second panel). Third panel: Western blot of total His_6_-Osh6p in the total yeast extract using anti-6xHis antibody. (B) Co-purification of 6xHis-tagged VAP27-1 (Scs2 ortholog) with the p33 and p92 replication proteins from subcellular membranes from yeasts with depleted Ufe1p (GALS::UFE1), depleted Use1p (GAL1::USE1) or WT yeasts. Top panel: Western blot analysis of co-purified His_6_-VAP27-1 protein with Flag-affinity purified FLAG-p33. His_6_-VAP27-1 was detected with anti-6xHis antibody, while FLAG-p33 was detected with anti-FLAG antibody (second panel). Third panel: Western blot of total His_6_-VAP27-1 in the total yeast extract using anti-6xHis antibody. (C-D) Interaction between TBSV nYFP-p33 replication protein and the Scs2-cYFP protein was detected by BiFC. Partial co-localization of RFP-Ufe1 with the BiFC signal (see merged image) demonstrates that the interaction between p33 replication protein and Scs2p VAP protein occurs in the vicinity of the Ufe1-positive subdomain of the ER membrane at 6 and 16 h after induction, respectively. (E) Confocal laser microscopy analysis shows the diminished interaction between the TBSV nYFP-p33 replication protein and the Scs2-cYFP protein (detected by BiFC) when the dominant-negative Ufe1ΔTM is co-expressed in wt yeast cells.

To gain insights into the pro-viral function of Ufe1p, we tested if Ufe1p and Use1p could affect the formation of TBSV-induced membrane contact sites (vMCS), which are involved in the enrichment of sterols at the sites of virus replication [43,44]. The oxysterol-binding proteins (OSBP-like, Osh proteins in yeast) and the ER-resident VAP protein (Scs2 in yeast and Vap27-1 in plants) are subverted by TBSV to form vMCS. Indeed, the less efficient co-purification of Osh6p and Vap27-1 with p33 replication protein from yeast co-expressing Ufe1ΔTM (Table 1) suggests less efficient formation of viral-induced vMCSs. First, we depleted Ufe1p and Use1p, respectively, by growing yeast on glucose media (based on GALS::UFE1 and GAL1::USE1 yeast strains, which express either Ufe1p from the *GAL1S* promoter or Use1p from the *GAL1* promoter, see Fig 3), followed by purification of the viral replicase from detergent-solubilized membrane fractions of yeasts expressing FLAG-p33 and FLAG-p92 replication proteins and the repRNA. Western blotting-based measurements of the co-purified cellular proteins revealed that VAP27-1 was present in ~5-fold reduced amount when either Ufe1p or Use1p were depleted in yeasts (Fig 7B). A similar approach revealed ~7-fold reduction in Osh6p amount in the purified replicase preparations obtained from yeast with depleted Ufe1p or Use1p (Fig 7A). Because VAP proteins and Osh proteins are critical for vMCS formation [43,44], the greatly reduced co-purification of VAP27-1 and Osh6p with the viral replication proteins from yeasts with depleted Ufe1p or Use1p strongly suggests the inefficient formation or reduced stability of vMCSs.

Second, we further tested the role of Ufe1p in vMCS formation by using BiFC with Scs2p VAP and p33 replication protein [43] to mark the TBSV-induced vMCSs in yeast. The co-expressed RFP-Ufe1p partially co-localized with Scs2-p33 complex at the 6 h time point (Fig 7C), and even at the 16 h time point when robust TBSV replication occurs in yeast (Fig 7D). Co-expression of Ufe1ΔTM greatly reduced the BiFC signal between Scs2p and p33 (Fig 7E), promoting the idea that Ufe1ΔTM inhibits the formation of TBSV-induced vMCSs in yeast.

**Fig 8:**
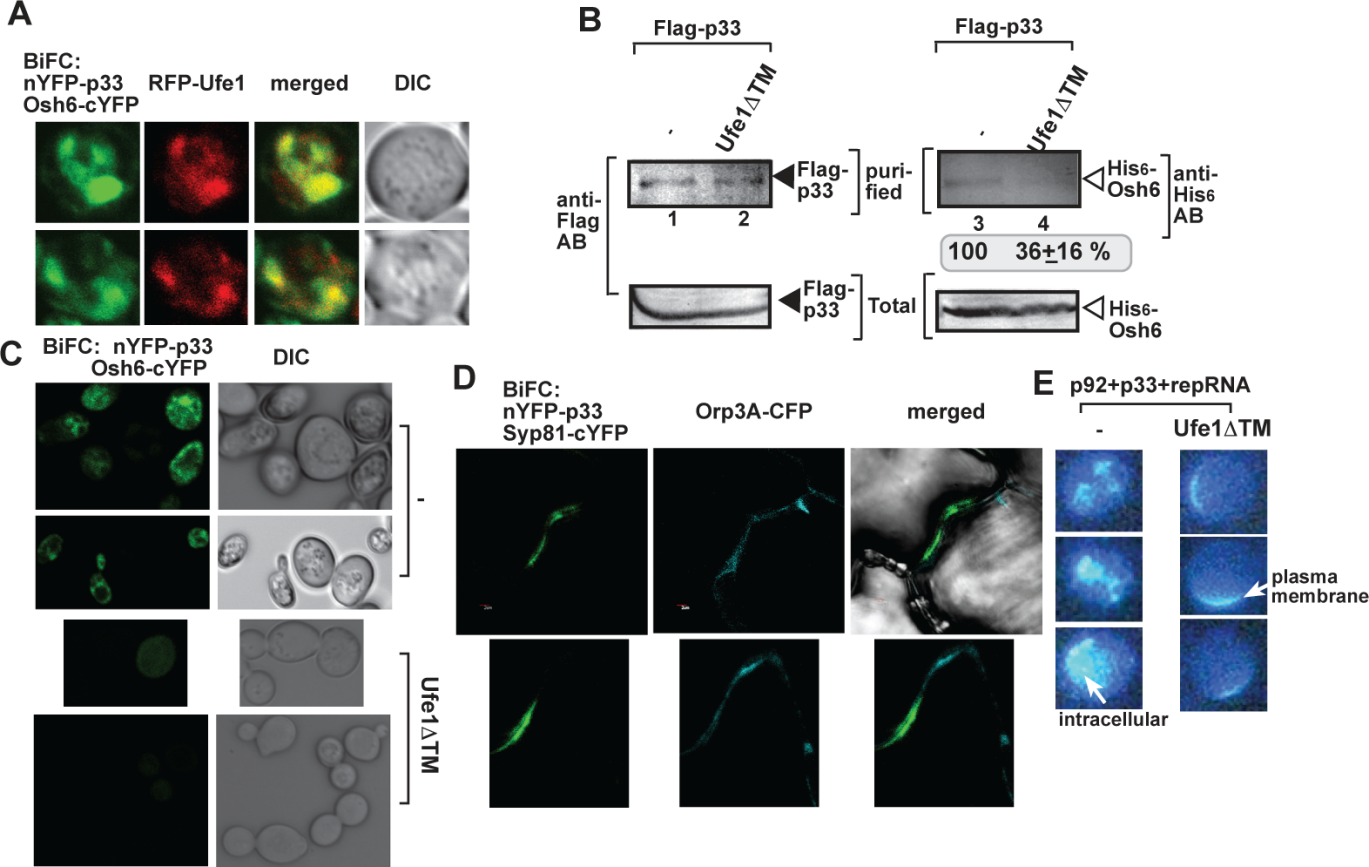
P33 replication protein interacts with Osh6p oxysterol-binding protein in the vicinity of Ufe1-positive membranes. (A) Interaction between of TBSV nYFP-p33 replication protein and the Osh6-cYFP protein was detected by BiFC. Partial co-localization of RFP-Ufe1 with the BiFC signal (see merged image) demonstrates that the interaction between p33 replication protein and Osh6p protein occurs in the vicinity of the Ufe1-positive subdomain of the ER membrane. The images were taken at the 16 h time point. (B) Co-purification of Osh6p with the p33 replication protein from subcellular membranes is inhibited by co-expression of the dominant-negative Ufe1ΔTM. Top panels: Western blot analysis of co-purified His_6_-tagged cellular Osh6p protein (lanes 3–4) with Flag-affinity purified FLAG-p33 (lanes 1–2) from detergent-solubilized membrane fraction of wt yeast. His_6_-Osh6p was detected with anti-His antibody, while FLAG-p33 was detected with anti-FLAG antibody. Note that yeasts co-expressed the dominant-negative Ufe1ΔTM (lanes 2 and 4). Bottom panels: Western blot of total HA-Ufe1p or HA-Use1p in the total yeast extract using anti-HA antibody. Bottom panel: Western blot of total FLAG-p33 and His_6_-Osh6p in the total yeast extracts. (C) Confocal laser microscopy analysis shows the diminished interaction between the TBSV nYFP-p33 replication protein and the Osh6-cYFP protein (detected by BiFC) when the dominant-negative Ufe1ΔTM is co-expressed in wt yeast cells. (D) Interaction between TBSV nYFP-p33 replication protein and the AtSyp81-cYFP protein (detected by BiFC) occurs at vMCS. Co-localization of ORP3A-CFP (small OSBP-like protein, ortholog of yeast Osh6p) with the BiFC signal (see merged image) demonstrates that the interaction between p33 replication protein and AtSyp81 in subdomains of the ER membrane likely facilitates vMCS formation. The proteins were expressed via agroinfiltration in *N*. *benthamiana*, followed by inoculation with TBSV. The images were taken 3 days latter. (E) Co-expression of the dominant-negative Ufe1ΔTM interferes with TBSV p33-driven sterol enrichment at internal replication sites. Sterols (ergosterols) were stained with filipin dye, followed by fluorescent microscopic analysis. Note that ergosterol are mostly present in the plasma membrane when dominant-negative Ufe1ΔTM was co-expressed with the viral components (right images), whereas sterols were present at internal viral replication sites in the absence of Ufe1ΔTM (images on the left).

To further test the putative role of Ufe1p in TBSV-induced vMCS formation, we took advantage of another major player, Osh6p, which is one of the members of oxysterol-binding protein family playing a role in TBSV-induced vMCS formation [43]. We detected the co-localization of Ufe1p with the p33 and Osh6p complex via BiFC (Fig 8A). Expression of Ufe1ΔTM in yeast reduced the amount of co-purified Osh6p with p33 replication protein from detergent-solubilized yeast membrane fraction by ~3-fold (Fig 8B). Similar to the above case with p33-Scs2p interaction, co-expression of Ufe1ΔTM also greatly reduced the BiFC signal between Osh6p and p33 (Fig 8C), further supporting the model that Ufe1p contributes to the formation of TBSV-induced vMCSs in yeast. We also observed co-localization among AtOrp3A (ortholog of the yeast Osh6) and p33 replication protein and AtSyp81 (ortholog of the yeast Ufe1), in *N*. *benthamiana* leaves (Fig 8D), indicating that AtSyp81 interacts with p33 in the vMCS in *planta*. Altogether, the emerging picture is that the ER resident Ufe1p is localized in a subdomain of ER that is subverted for vMCS formation.

The major role of the TBSV-induced vMCSs is the enrichment of various sterols at the site of replication to facilitate tombusvirus replication [43,45]. Accordingly, expression of Ufe1ΔTM in yeast replicating TBSV repRNA reduced the enrichment of sterols at internal locations (Fig 8E), which have been shown to represent replication compartments [43]. Instead, sterols mostly accumulated in the plasma membrane in yeast expressing Ufe1ΔTM. Because sterols are the most abundant in the plasma membrane in yeast lacking TBSV components [43], we suggest that TBSV could not efficiently recruit sterols to the intracellular replication compartments when Ufe1ΔTM inhibited vMCS formation. Based on all these data, we suggest that Ufe1p-p33 interaction is important for vMCS formation and the enrichment of sterols within the viral replication compartments.

**Fig 9:**
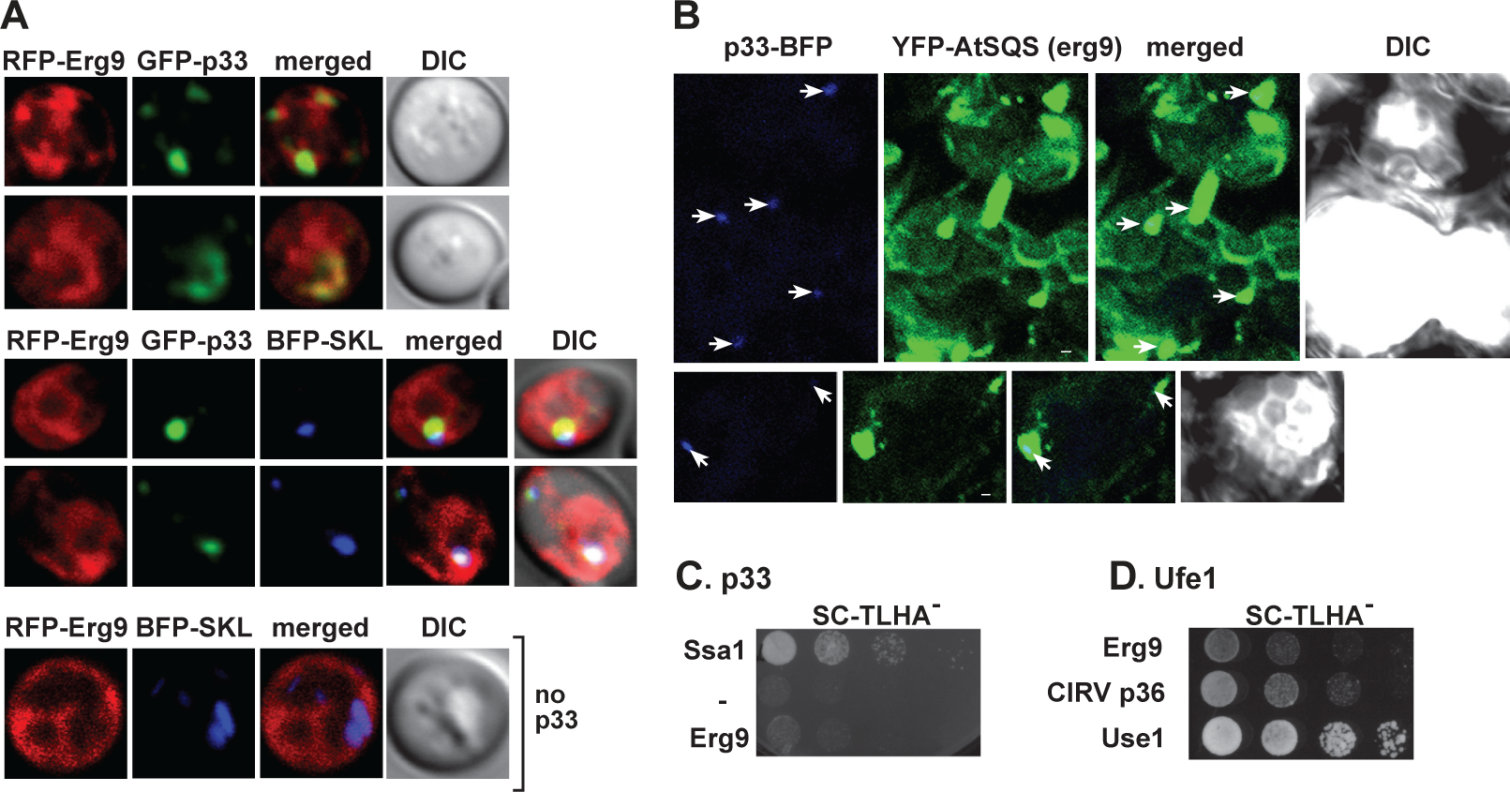
Co-localization of p33 replication protein with sterol biosynthesis proteins in yeast and plant cells. (A) Top two images: Confocal laser microscopy analysis of subcellular distribution of GFP-tagged p33 and RFP-Erg9 in wt yeast cells. Middle images: Confocal laser microscopy analysis of subcellular distribution of GFP-tagged p33, RFP-Erg9 and BFP-SKL, which marks the peroxisomes, in wt yeast cells. Bottom image: proximal localization of RFP-Erg9 and BFP-SKL in wt yeast. (B) Confocal microscopic images show that p33-BFP (marked by arrows) is localized within subcellular membranes also harboring YFP-tagged SQS1, which is the plant homolog of Erg9, and is involved in sterol biosynthesis. Expression of the above proteins from the 35S promoter was done after co-agro-infiltration into *N*. *benthamiana* leaves. Scale bars represent 2 μm. (C) Lack of interaction between the TBSV p33 replication protein and Erg9 based on the split ubiquitin assay. The bait p33 was co-expressed with N-terminally-tagged Erg9p protein. *SSA1* (HSP70 chaperone), and the empty prey vector (NubG) were used as positive and negative controls, respectively. (D) Interaction between the yeast Ufe1p SNARE protein and Erg9 based on the split ubiquitin assay. The bait Ufe1p was co-expressed with N-terminally-tagged Erg9p protein. The split ubiquitin assay shows interaction between the yeast Ufe1p and the CIRV p36 replication protein and the yeast Use1p SNARE protein, respectively. Find further details in the legend of Fig 1.

Sterols (ergosterols in yeast) are synthesized in the subdomains of ER, and Erg9 enzyme, which is squalene synthase, a critical enzyme in sterol biosynthesis, is known to interact with Ufe1p in the ER [46]. This highlights the possibility that Ufe1p (and thus ERAS subdomain) might be targeted for hijacking by TBSV to facilitate the rapid sterol enrichment within the replication compartment. Indeed, p33 was localized in the close vicinity of Erg9p in yeast cells (Fig 9A, top panels). The peroxisomes subverted by TBSV for replication compartments were also localized in the vicinity of ER-resident Erg9p (Fig 9A, middle panels). Similar observations could be drawn from plant cells co-expressing p33-BFP and YFP-SQS1 squalene synthase (Fig 9B), the plant ortholog of Erg9p [47].

However, Erg9p does not interact directly with p33 in the MYTH assay (Fig 9C), but Erg9p interacts with Ufe1p (Fig 9D) [46]. All these results suggest that sterol biosynthesis likely occurs in the vicinity of p33-Ufe1p complex that might facilitate the rapid subversion of the newly and locally synthesized sterols into the viral replication compartments. Further experiments will be needed to test if additional cellular sterol producing proteins are also localized in the vicinity of TBSV replication sites.

### Ufe1—p33 complexes are present in ER subdomains targeted by the p33-driven Rab5-positive endosomes needed for replication compartment biogenesis in yeast

**Fig 10:**
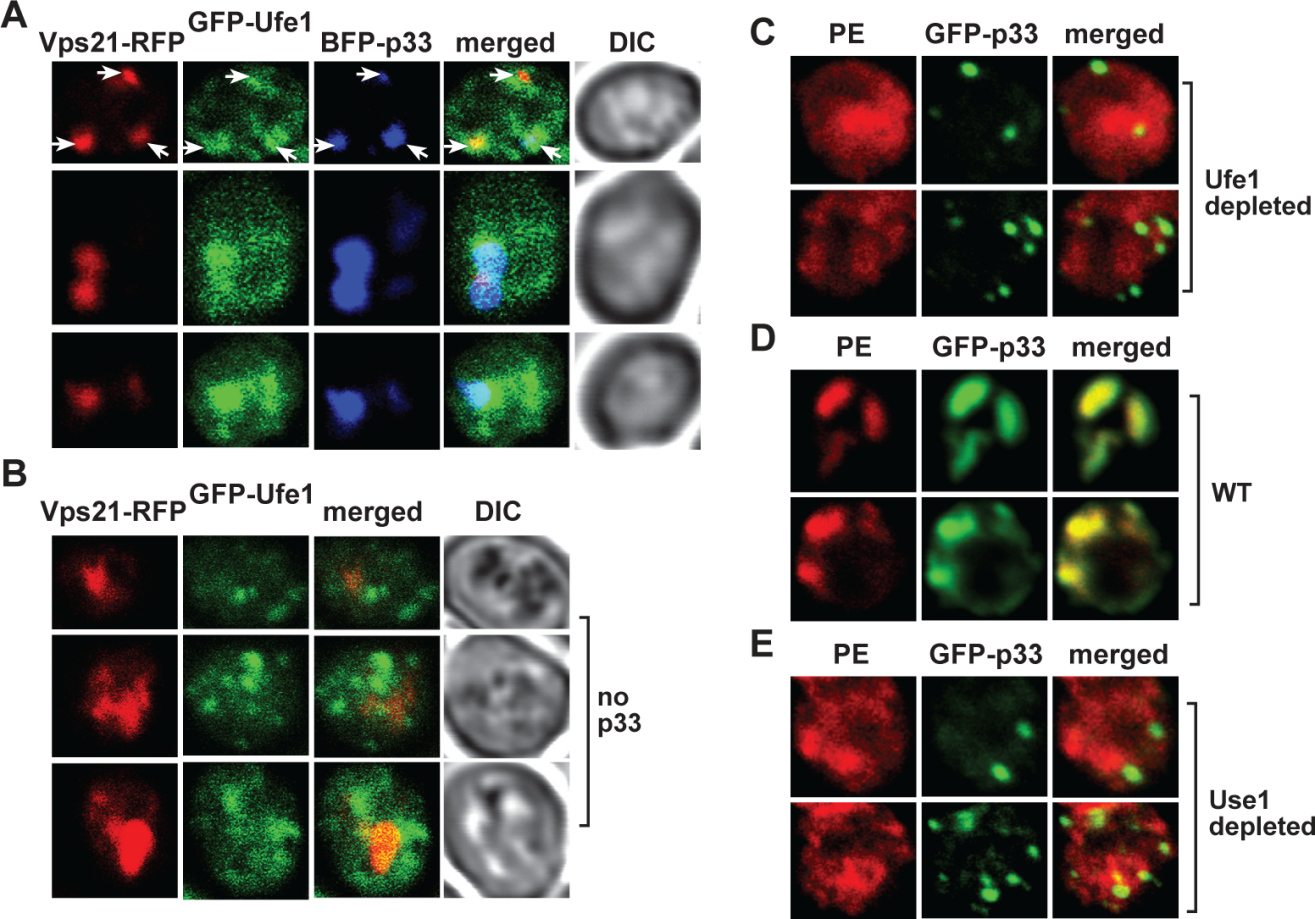
The role of the ER SNARE proteins in enrichment of PE in the viral replication compartment in yeast cells. (A) Confocal laser microscopy images show the partial co-localization of TBSV BFP-tagged p33 replication protein with the RFP-tagged Vps21p protein and GFP-Ufe1p SNARE protein in wt yeast cells. DIC (differential interference contrast) images are shown on the right. Arrows point at sites enriched for GFP-Ufe1p in subcellular locations also containing BFP-p33 and RFP-Vps21p. (B) Confocal laser microscopy images show the different localization pattern for RFP-tagged Vps21p protein (early endosomes) and GFP-Ufe1p SNARE protein (ER subdomains) in wt yeast cells. (C) Confocal laser microscopy images show the lack of enrichment of PE in the BFP-p33 replication protein-positive replication compartments in Ufe1p repressed yeast cells (“Ufe1 kd”, top two images). GALS::UFE1 yeast cells were grown in glucose media for 16 h. Localization of PE is detected by using biotinylated duramycin peptide and streptavidin conjugated with Alexa Fluor 405. (D) Control confocal laser microscopy images show the enrichment of PE within the BFP-p33 replication protein-positive replication compartments in wt yeast cells. See further details in Panel C. (E) Confocal laser microscopy images show the poor level of enrichment of PE in the BFP-p33 replication protein-positive replication compartments in Use1p repressed yeast cells (“Use1 kd”). GAL1::USE1 yeast cells were grown in glucose media for 16 h. See further details in Panel C.

In addition to sterol enrichment, the formation of the TBSV replication compartment also depends on the retargeting of Rab5-positive endosomes, which are PE-rich, to the growing viral replication compartments [48]. Here, we used confocal microscopy to show that the Rab5 (called Vps21 in yeast)-positive endosomes were co-localized with GFP-Ufe1 and BFP-p33 in yeast cells (Fig 10A), supporting the idea that the ER subdomain containing Ufe1p is the site of the biogenesis of the tombusvirus replication compartment. As expected based on the known endosomal Vps21p and ER localization of Ufe1p, these proteins showed different localization pattern in yeast lacking viral components (Fig 10B).

The TBSV replication compartment is PE-enriched [49] based on subversion of Rab5-positive endosomes [48]. To test if Ufe1p affects the enrichment of PE within the replication compartment, we depleted Ufe1p level in yeast, followed by subcellular localization of PE and GFP-p33 via confocal microscopy. These experiments revealed the lack of PE enrichment in p33-decorated viral replication compartment when Ufe1p was depleted (Fig 10C), whereas the PE was efficiently enriched in the replication compartment in wt yeast (Fig 10D) [49]. Depletion of Use1p also inhibited PE enrichment in the replication compartment (Fig 10E). Based on these results, we suggest that Ufe1 and Use1 SNARE proteins are involved in recruitment of the PE-rich Rab5-positive endosomes into the viral replication compartment.

#### Retrograde vesicle transport from the Golgi-to-ER is not required for TBSV replication

**Fig 11:**
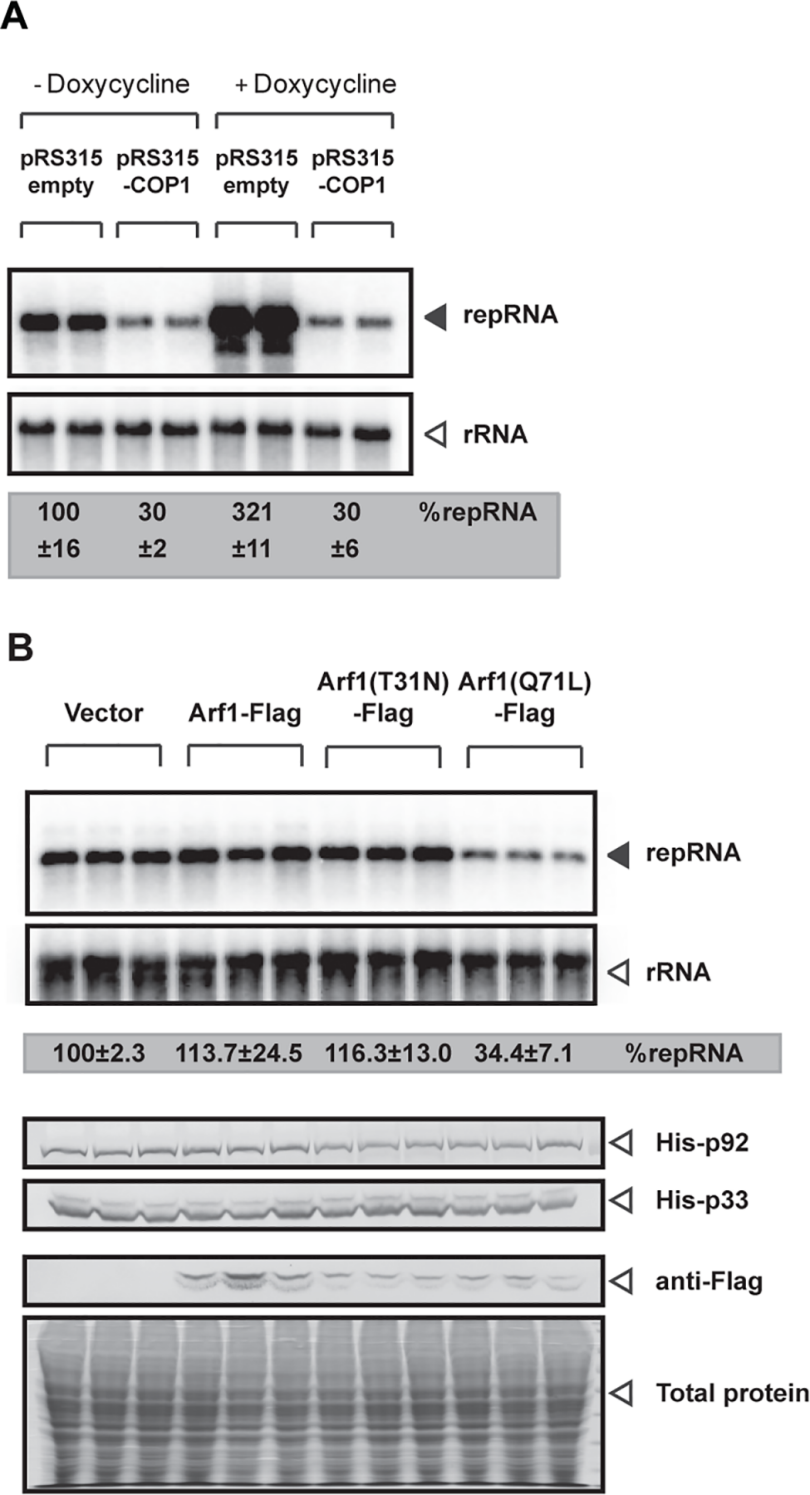
*COP1* vesicular transport gene is not required for TBSV repRNA accumulation in yeast. (A) Northern blot analysis of TBSV repRNA shows increased accumulation of repRNA in THC-COP1 yeast strain when Cop1 level is repressed. Viral proteins His_6_-p33 and His_6_-p92 were expressed from plasmids from the copper-inducible *CUP1* promoter, while DI-72(+) repRNA was expressed from the galactose-inducible *GAL1* promoter. The expression of *COP1* was repressed by doxycycline (+ samples) or induced by omitting doxycycline (“-”samples) from the yeast growth media. Cop1p was expressed from the constitutive *TEF1* promoter (lanes 3–4 and 7–8). Bottom image: Northern blot with 18S ribosomal RNA specific probe was used as a loading control. Each experiment was repeated. (B) Northern blot analysis of TBSV repRNA shows that expression of dominant-active mutant of Arf1p small GTPase inhibits tombusvirus replication in yeast. The WT FLAG-tagged Arf1p, the dominant-negative mutant T_31_N (i.e., the GDP-bound form) and the dominant-active mutant Q_71_L (i.e., the GTP-bound form), respectively, were expressed from the inducible *CUP1* promoter in wt yeast cells. Middle and bottom panels show Western blot analysis of TBSV p33, p92 and Arf1-FLAG accumulation level.

The canonical function of Ufe1p/Use1p/Sec20p ER SNAREs is to form the ER arrival site (ERAS), which participates in the COP-I-mediated retrograde vesicle transport from the Golgi-to-ER [29,30,31,32]. To test if the ER SNARE proteins could also affect TBSV replication indirectly through the retrograde transport from Golgi-to-ER, we down-regulated the alpha subunit of the COP-I vesicle coatomer complex, namely Cop1p, by adding doxacycline to the yeast strain THC-COP1, in which the natural promoter of *COP1* is replaced with the Tet-regulatable promoter [50]. Interestingly, we found that TBSV repRNA accumulation is increased by ~3-fold when *COP1* expression was down-regulated in comparison with TBSV repRNA accumulation when yeast expressed Cop1p (Fig 11A). In addition, over-expression of Cop1p under a constitutive promoter from a plasmid (pRS315-COP1) inhibited TBSV repRNA accumulation by ~70% (Fig 11A), suggesting that Cop1p or the formation of COP-I vesicle coatomer complex interferes with TBSV replication.

To further test the role of the canonical function of ER SNAREs in TBSV replication, we took advantage of the dependence of formation of the COP-I vesicles on Arf1p small GTPase [51]. Briefly, we expressed mutants of Arf1p, including dominant-negative mutant Arf1-T_31_N and dominant-active mutant Arf1-Q_71_L, which encodes a GTP-locked form of Arf1p. The dominant-negative mutant Arf1-T_31_N blocks the formation of COP-I vesicles, and interferes with COP-I-dependent retrograde transport. On the contrary, dominant-active Arf1-Q_71_L stimulates the formation of COP-I vesicles coats, but blocks coatomer disassembly at the ER [51,52]. Over-expression of wt Arf1p or expression of dominant-negative Arf1-T_31_N did not alter TBSV repRNA accumulation in comparison with yeast transformed with an empty plasmid. On the other hand, expression of dominant-active Arf1-Q_71_L reduced replication by ~65% (Fig 11B). Viral replication protein accumulation in Arf1-Q_71_L-expressing yeast showed similar level to that found in control, Arf1p or Arf1-T_31_N-expressing yeasts (Fig 11B). Based on these results, it seems that the COP-I-mediated retrograde transport is not required for TBSV replication. Furthermore, because dominant-active Arf1-Q_71_L blocks COP-I coatomer disassembly at the ER [51,52], we suggest that the canonical function of Ufe1p/Use1p/Sec20p ER SNAREs in ERAS competes with the co-opted functions of Ufe1p/Use1p/Sec20p in TBSV replication.

## Discussion

Formation of the (+)RNA virus-induced extensive replication compartments in infected cells is incompletely understood. In case of TBSV, which replicates on the boundary membranes of peroxisomes in yeast and plant cells, the replication process leads to the formation of large, multivesicular body-like structures containing dozens or even hundreds of individual spherules harboring VRCs [14,53,54]. The role of Pex19p peroxisome membrane-biogenesis protein in the formation of TBSV replication compartment, however, is not essential, because TBSV could replicate in yeast cells missing peroxisomes [18]. In the peroxisome-free yeast cells, TBSV utilizes the ER membranes for efficient replication [21,27], suggesting that the ER membrane likely contains pro-viral cellular proteins assisting VRC assembly. Accordingly, in this work, we find major roles for the ER-resident Ufe1p and Use1p SNARE proteins and the ERAS subdomain of ER in the formation of TBSV replication compartment as discussed below.

### Ufe1 and Use1 SNARE proteins are pro-viral host factors in tombusviral replication

Co-purification, MYTH, BiFC and subcellular co-localization results revealed that syntaxin-18-like Ufe1p and Use1p SNARE proteins interact with the TBSV p33 replication protein. Depletion of Ufe1p or expression of the dominant negative mutant of Ufe1p in yeast, which inhibits Ufe1p functions, inhibited TBSV repRNA accumulation in yeast and viral replication in *N*. *benthamiana*. The delayed expression of the dominant negative mutant of Ufe1p did not significantly inhibit TBSV replication, or the replication compartment formation, suggesting that the p33-Ufe1p interaction likely functional during the early steps of the replication process. Accordingly, in vitro reconstitution of the tombusvirus replicase in CFEs from yeast with depleted Ufe1 or Use1 levels showed defect in the replicase assembly process. Ufe1p was also required for TBSV replication in peroxisome-free yeast, suggesting that Ufe1p-p33 interaction and, thus, the ER membrane is more critical for TBSV replication than the peroxisome membrane. Similarly, depletion of Use1p had inhibitory effect on TBSV repRNA in yeast.

A closely related tombusvirus, CIRV, which replicates on the mitochondrial outer membrane, also co-opts Ufe1/Syp81 syntaxin through direct interaction between p36 replication protein and Ufe1p. In addition, expression of the dominant negative mutant of Syp81 inhibited CIRV RNA accumulation in *N*. *benthamiana*. Altogether, these observations provide strong evidence that Ufe1/Syp81 syntaxin and Use1 ER SNARE proteins have important pro-viral functions.

### Ufe1 and Use1 SNARE proteins are involved in the biogenesis of the tombusviral replication compartments in cells

Deciphering the actual pro-viral functions of Ufe1p and Use1p is a challenge due to the multifunctional nature and essential roles of these proteins in yeast viability. The best-known functions of Ufe1p and Use1p are performed as part of the ERAS/ERIS subdomain in the ER where the COP-I vesicles coming from the Golgi compartment are fused to the ER [29,30,31,32,33,55]. However, the ERAS/ERIS subdomain and the Dsl1 tethering complex also play a role in the generation of peroxisomal membrane in yeast [35]. Therefore, Ufe1p and the ERAS/ERIS subdomain might be co-opted by tombusviruses to induce or facilitate the membrane biogenesis required for building the large viral replication compartments. Accordingly, p33 interacts with Ufe1p and Use1p in a special subdomain of ER, which is localized in the vicinity of p33-positive peroxisomes. Blocking the function of the ERAS/ERIS subdomain through depletion of Ufe1p and Use1p, down-regulation of Sec20p (S2C Fig) or inhibition of the Dsl1 tethering complex through depletion of Sec39p [28] and expression of Dsl1p or Tip20p mutants (S2D–S2F Fig) led to greatly reduced TBSV RNA accumulation in yeast or poor viral replicase activities *in vitro* in a replicase reconstitution assay. These observations suggest that the Ufe1p-positive ERAS subdomain in ER is involved in the biogenesis of the TBSV replication compartment.

Interestingly, the membranes used by TBSV for replication have modified lipid contents that are enriched in PE and sterol lipids [43,49]. Ufe1p is frequently co-localized with Scs2p VAP-p33 and Osh6p-p33 protein complexes, the known members of TBSV-induced vMCSs, which suggests that those vMCSs between the ER and peroxisomes are formed in the proximity of the Ufe1-positive ER subdomains. The p33-containing vMCSs have been suggested to facilitate the local enrichment of sterols within the replication compartment [43]. Expression of the dominant-negative Ufe1p interfered with the p33-induced re-localization of ergosterol in yeast cells (Fig 8E), and also inhibited the complex formation between Scs2p VAP protein and p33 (Fig 7E) and Osh6p ORP and p33 (Fig 8B). Depletion of either Ufe1p or Use1p also greatly inhibited the complex formation between VAP27-1 and p33 and Osh6p ORP and p33 (Fig 7). All these data suggest that Ufe1p-positive ERAS subdomains are involved in the formation of TBSV-induced vMCSs. Moreover, Ufe1p might further facilitate ergosterol enrichment at replication sites by interacting with the ER-resident Erg9 ergosterol biosynthesis enzyme, which indeed was localized near the replication compartment in yeast or the orthologous SQS1 in plant cells infected with TBSV (Fig 9).

**Fig 12:**
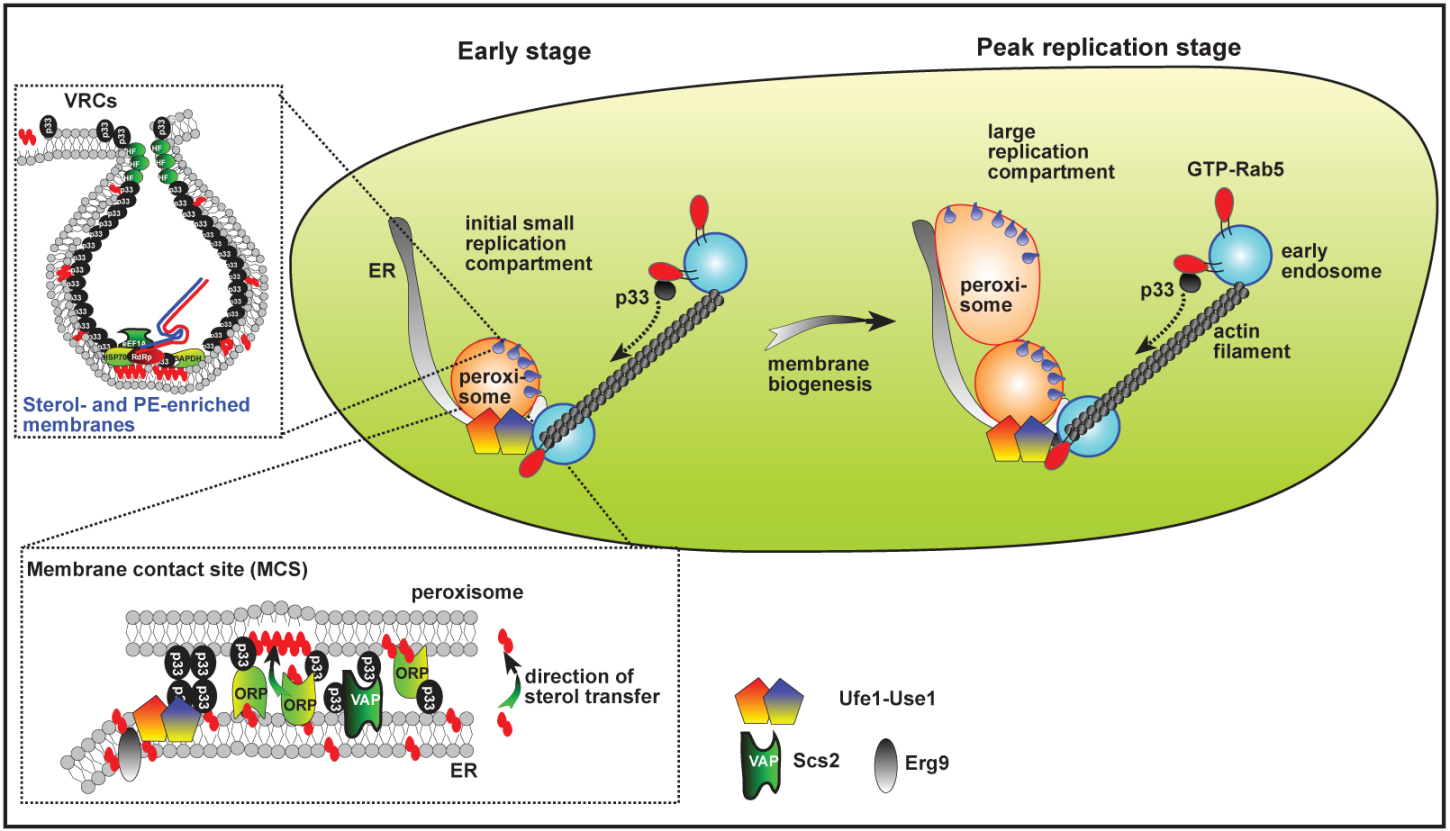
A model on the assembly hub role of Ufe1 and Use1 SNARE proteins and the ERAS subdomain in ER in the formation of the TBSV replication compartment. Early stage of replication: Through direct interactions between p33 replication protein and Ufe1p and between p33 and Use1, TBSV pre-assemble protein complexes at membrane contact sites (vMCS) formed between the ER and a peroxisome. Co-opted cellular proteins, Ufe1, Use1 SNAREs, Erg9, Scs2 VAP and Osh6 ORP and the viral p33 replication proteins located at vMCS, are shown in the dotted box. Enrichment of sterols and PE and recruitment of additional cellular proteins allows the formation of replication compartment harboring spherules (purple). Ufe1- and Use1-positive ERAS subdomain in the ER facilitates the recruitment of Rab5-positive endosomal vesicles to the replication compartment, leading to PE enriched membranes and enlargement of the replication compartment. Hijacked actin filaments likely facilitate the efficient enlargement of the replication compartment [48,76]. Peak stage of replication: The enlarged replication compartment with numerous spherules. See main text for further description.

Another source of lipids and membranes exploited by tombusviruses is the Rab5-positive endosomes, which are PE-rich [48]. We found that Rab5 (yeast Vps21p) and p33 replication protein are co-localized with Ufe1p in yeast cells (Fig 10), suggesting that TBSV co-opts Rab5-positive endosomes to the Ufe1/Use1 ERAS subdomain. Moreover, depletion of either Ufe1p or Use1p inhibited the enrichment of PE at replication sites, indicating that the Ufe1/Use1 ERAS subdomain is required for PE-enrichment in the viral replication compartment. Based on these observations, Ufe1/Use1-positive ERAS subdomains might serve multiple pro-viral functions such as pre-assembly sites for VRC formation, p33-positive peroxisome membrane biogenesis, as well as for sterol- and PE-enrichment in the tombusvirus replication compartment (Fig 12). Overall, our studies have established that components of the ERAS/ERIS subdomain of ER, including Ufe1p and Use1p SNAREs and the Dsl1 tethering complex, are critical for robust and efficient TBSV replication in yeast and plant cells.

The pro-viral function of Ufe1p during the biogenesis of the tombusvirus replication compartment might be similar (to a certain degree) to the recently uncovered noncanonical function of Ufe1p in biogenesis of the autophagosomal membranes. It has been suggested that direct transfer of ER membranes from specific ER subdomains containing Ufe1p could occur to sites of autophagosome formation [56]. In addition, cellular transport vesicles, including endosomal vesicles, can also be diverted to promote autophagosome formation in Rab-GTPase dependent manner. Ufe1p working together with endosomal SNAREs might function during autophagosome formation by promoting membrane fusion that could provide the membrane supply for the double membrane autophagosomes [56,57]. This process is also Sey1p independent, similar to the biogenesis of the tombusvirus replication compartment.

Subcellular localization studies with p33 replication protein revealed that p33 mislocalized to the ER in yeast under limited Ufe1p expression. The peroxisome biogenesis is hindered under this condition (Fig 4) due to the essential roles of ERAS/ERIS and the Dsl1 tethering complex in peroxisome membrane organization [35]. Based on our observations, we suggest that Ufe1p and Use1p cellular proteins might play a direct and a central role in TBSV replication by anchoring the TBSV replication proteins and several co-opted host proteins to specific ERAS subdomains, which might provide the initial membrane domains for the pre-assembly of the tombusvirus replicase complexes. It is unlikely that Ufe1p or Use1p are involved in recruitment of host proteins (other than the sterol synthesis proteins), but rather serve as an organization hub to build the replication compartment and that stabilizes the interaction of p33 with several co-opted host factors.

The pro-viral function of Ufe1p seems to be in competition with the canonical cellular function of Ufe1p in ERAS, which serves as a docking site for the COP-I vesicles coming from the Golgi. Accordingly, over-expression of Cop1p alpha subunit of COP-I coatomer complex or expression of the dominant-active Arf1p small GTPase, which blocks disassembly of COP-I coatomer at ERAS, inhibited TBSV replication in yeast. On the contrary, down-regulation of Cop1p, which reduces COP-I vesicle formation, enhanced TBSV replication in yeast. These data also exclude the model that down-regulation of Ufe1p or Use1p in yeast indirectly reduces TBSV replication due to limiting retrograde vesicle transport from Golgi to ER. Interestingly, Arf1 is exploited by several (+)RNA viruses, such as polio, hepatitis C and Dengue viruses and red clover necrotic mosaic virus for replication and other purposes [58,59,60,61,62].

Overall, the results presented in this paper suggest a major pro-viral function for Ufe1p and Use1p SNARE proteins and ERAS in the formation of the extensive tombusvirus replication compartment by supporting membrane proliferation, which are then exploited by tombusviruses.

### Ufe1 and Use1 SNARE proteins, which are present in active ERAS subdomains, serve as TBSV replicase assembly hubs

So why do TBSV (peroxisomal replication) or CIRV (mitochondrial replication) prefer the Ufe1/Use1 ERAS subdomain for initial replicase assembly? Recent works suggest that Ufe1/Use1 containing ERAS/ERIS subdomains are very active in many processes. For example, ERAS/ERIS subdomains serve as places/hubs for COP-I vesicle arrival, intense membrane-expansion, robust material exchange, some membrane proteins and sterol transfer involving lipid droplets and they associate with active actin patches [55,63,64]. We propose that sequestering and concentrating the viral replication proteins and several co-opted host proteins within limited ERAS subdomains in the otherwise vast ER membranes could facilitate protein-protein interactions, lipid enrichment, and more robust VRC assembly process. By targeting Ufe1p and Use1p SNARE proteins, TBSV might also take advantage of the functions of these proteins to generate new membranes, which could then be hijacked by TBSV for the formation of large replication compartments (Fig 12).

Interestingly, the unrelated brome mosaic virus (BMV) is proposed to target the ER exit sites (ERES), which are ER subdomains where the COP-II coated vesicles leave the ER to Golgi, for viral replicase assembly [65]. Thus, these very active ER subdomains, i.e., ERAS/ERIS in case of TBSV and ERES for BMV, might be especially suitable for generation of the membranous viral replication compartments.

Various SNARE proteins have been shown to affect different viruses and multiple steps in the infection cycle. For example, Syp71 SNARE protein is recruited to aid turnip mosaic virus replication via mediating membrane fusion events between virus-induced vesicles and the chloroplasts [66,67]. Syntaxin 17, an autophagosomal SNARE protein, and syntaxin 4, a SNARE involved in intracellular vesicle traffic, have been shown to affect the release of HCV virions from infected cells [68,69]. Similarly, human parainfluenza virus type 3 inhibits the function of Syntaxin 17 to block autophagy and boost extracellular viral production [70]. The human pathogen, BK polyomavirus utilizes syntaxin-18 (Ufe1 in yeast) for intracellular trafficking from the endosomes to the ER [71]. Syntaxins are also involved in cellular trafficking by DNA viruses [72,73,74]. Silencing of syntaxin 5, a Golgi SNARE, inhibited the reverse transcription step in HIV infection [75]. Altogether, all these works support the emerging significance of co-opted SNARE proteins during viral infections.

## Materials and methods

### Yeast strains and expression plasmids

*Saccharomyces cerevisiae* strains BY4741 and R1158 (wt) [50] were obtained from Open Biosystems. See further details in the supplementary materials (S1 Text).

### Confocal laser microscopy analysis

Co-localization and Bimolecular Fluorescence Complementation Assay in yeast. WT BY4741 (Open Biosystems) strain was transformed with LpGAD-ADH1-RFP-6xHis-ScUfe1 along with UpYES-GAL1-GFP-p33. Transformed yeast was grown in UL^-^ minimal media supplemented with 2% glucose for 12 hours and then yeast cells were harvested, washed and media was changed to UL^-^ minimal media supplemented with 2% galactose to induce p33 expression. Cells were harvested and washed after 6, 16, 24 and 36 h. Then 2–3 µl of cell suspension was dropped on poly-L-lysine coated slides and visualized with Olympus FV1000 laser scanning microscope [76]. Single frames are obtained.

To examine the subcellular localization of Vps21 and Ufe1 in the presence and absence of the viral replication protein, Ufe1 was tagged with GFP on the chromosome, replacing the WT *UFE1*. Primers F6198/R6199 and plasmid pYM-N33 (EUROSCARF plasmid collection) were used to create strain BY4741 UFE1::GALS-GFP-Ufe1-NatNT2 according to authors’ recommendation [77]. Then the above yeast strain was transformed with plasmids LpRS315-TEF1-RFP-Vps21 and HpESC-GAL1-BFP-p33 [48] or HpESC empty as a control. Transformed yeast cells were grown on LH^-^ SC media containing 1% galactose and 1% raffinose for 16 h at 23°C. Then confocal images were taken [76].

### Co-localization of p33-GFP and RFP-Syp81 in *N*. *benthamiana* protoplast

*N*. *benthamiana* protoplasts were co-electroporated with 5 µg plasmid DNA encoding for RFP–Syp81 (pGD-35S-RFP-Syp81) and 5 µg plasmid DNA encoding p33-GFP (pGD-35S-p33-GFP). Preparation of protoplasts were described earlier [78]. A total volume of 300 µl of the obtained protoplasts was pipetted into a 1 ml electroporation cuvette and mixed with an appropriate amount of mixtures of plasmids previously dissolved in 100 µl of electroporation buffer. The protoplasts were electroporated with Gene Pulser II set to 0.5 µF capacity and charge at 200 V. After 30 min of absolute rest, electroporated protoplasts were removed from the cuvettes and transferred to 2 cm Petri dishes with 600 µl of protoplast culture medium (PCM). Protoplasts were then incubated for 1.5 days at 25°C in a dark chamber. Then protoplasts were fixed with 3.7% formaldehyde in PCM [78], and then applied to poly-l-lysine–coated slides. Protein localization was detected with confocal laser scanning microscope using 488 nm laser for GFP, and 543 nm for RFP.

### Testing the effect of depletion of Ufe1 and Use1 on TBSV replication in yeast

BY4741-based UFE1::GALS-3xHA-Ufe1-Nat and USE1::GAL1-3xHA-Use1-Nat yeast strains expressing 3xHA-tagged Ufe1 and 3xHA-tagged Use1, respectively, were transformed with HpGBK-CUP1-6xHis-p33/ADH1-DI72 and LpGAD-CUP1-6xHis-p92 and spread on LH^-^ agar plates supplemented with 1% raffinose and 1% galactose. When colonies appeared, then single colonies were streaked on LH^-^ agar plates supplemented with 1% raffinose and 1% galactose. To deplete the cells for Ufe1 or Use1, the above yeast strains were grown in 3 ml LH^-^ minimal media supplemented with 2% glucose and BCS (to inhibit virus replication) for 12 h at 23°C. Then, yeast cultures were harvested and washed. Aliquots of 1.5 ml culture were grown in a fresh LH^-^ minimal media supplemented with 2% glucose and BCS and other aliquots of 1.5 ml were grown in LH^-^ minimal media supplemented with 2% galactose for 12 h to regain Ufe1 and Use1 expression. Then, yeast cells were harvested, washed and re-suspended in fresh LH^-^ minimal media containing 50 μM CuSO_4_ to launch virus replication. Yeast cells were grown for 24 h at 23°C and then harvested and total RNA was extracted. TBSV repRNA accumulation was detected by Northern blot analysis. Expression levels of Ufe1 and Use1 were analyzed with anti-HA antibody followed by alkaline phosphatase-conjugated anti-rabbit antibody and detection with NBT-BCIP. The expression level of His_6_-tagged p33 was analyzed with anti-His-antibody followed by anti-mouse antibody conjugated to alkaline phosphatase.

To analyze subcellular distribution of p33 in the Ufe1 depleted yeast, HpESC-CUP1-CFP-p33 and LpGAD-ADH1-pho86-CFP were co-transformed into BY4741-based UFE1::GALS-3xHA-Ufe1-NatNT2. Growing conditions were similar as above with the exception that p33 expression was induced only for 12 h, then confocal laser imaging was performed using Olympus FV1000 microscope.

### TBSV replication in the presence of dominant negative Syp81 mutant in *planta*

Plasmids pGD-35S-Syp81ΔTM or pGD empty were transformed into Agrobacterium strain C58C1Rif. Four weeks old *N*. *benthamiana* leaves were agro-infiltrated (OD_600_ value 0.5) for the transient expression of the tagged protein. Leaves were agro-infiltrated with the empty pGD vector as a control. After 1.5 days, leaves were sap inoculated with TBSV or CIRV. Total RNA was extracted from the inoculated leaves 2 days after inoculation and from the systemic leaves 4 days after inoculation and then Northern blot analysis was performed [79]. Pictures were taken at 4 days post inoculation. Detection of Syp81ΔTM mRNA was performed by RT-PCR with primers #5880/#5533 on cDNA created by reverse transcription. Tubulin mRNA-specific RT- PCR reaction (primers #2860/#2859) were performed as control.

#### Analysis of TBSV replication in yeast cell-free extracts

Cell-free extracts (CFE) from yeast strains BY4741, BY4741-based UFE1::GALS-3xHA-Ufe1-NatNT2, BY4741-based USE1::GAL1-3xHA-Use1-NatNT2, BY4741-based dsl1m1 (S1 Text), TET::SEC20 and its corresponding WT R1158 strains were prepared as described previously [80] with the exceptions that yeast strains harboring GAL1 promoter-regulated gene expression were grown in 1% galactose and 1% raffinose containing media, whereas Sec20 expression was down-regulated by addition of 20 µg/ml doxycycline to the culture media. The MBP-tagged p33 and p92 proteins were purified from *E*. *coli* as described [81]. CFE-based reaction mixtures containing 0.1 µg of each purified protein, 0.5 µg *in vitro*-transcribed repRNA and CFE preparations in a 20 µl final volume were incubated at 25°C for 3 h. The amount of newly synthesized repRNA was analyzed in polyacrylamide/urea gels as described [82].

### Silencing of *SYP81* in *N*. *benthamiana*

Virus induced gene silencing (VIGS) in *N*. *benthamiana* was performed as described earlier [83]. To create the VIGS vector (pTRV2-*Nt*SYP81), a 449-bp cDNA fragment of *Nicotiana tabacum* syntaxin-81-like mRNA (NCBI Sequence ID: XM_016656530.1) was RT-PCR-amplified from a total RNA extract using the primer pairs: #5400 and #5401 harboring restriction sites *BamH*I and *Xho*I respectively and ligated to the pTRV2 plasmid digested with BamHI and *Xho*I. Silencing of the target gene was confirmed with primers #5402/#5403 on total RNA extract of pTRV2-*Nt*SYP81 and pTRV2_C-GFP_ agroinfiltrated plants. Further details can be found in the supplementary material (S1 Text).

## Supporting information

S1 Fig Proximal localization of peroxisomes with Ufe1-positive subdomain of ER. (A) Confocal microscopy images show the proximal localization of BFP-SKL (peroxisomal luminar marker) and RFP-Ufe1 ER SNARE protein in yeast lacking tombusviral components. (B) Confocal microscopy images show both proximal localization and co-localization of GFP-p33 and RFP-Ufe1 in wt yeast cells.
(TIF)

S2 Fig Repression of the expression of Sec20p ER SNARE protein inhibits TBSV repRNA accumulation in yeast. (A) Schematic representation of the ER SNARE complex (ERAS/ERIS subdomain of ER) with the bound dsl1-tethering complex containing three proteins. (B) Northern blot analysis of TBSV repRNA shows the reduced accumulation of repRNA in THC-SEC20 yeast strain. Viral proteins His_6_-p33 and His_6_-p92 were expressed from plasmids from the copper-inducible *CUP1* promoter, whereas DI-72(+) repRNA was expressed from the galactose-inducible *GAL1* promoter. TBSV replication was induced by growing yeast cells in media containing 2% galactose (also 2% raffinose as a carbone source) and 50 μM CuSO_4_ at 23°C for 36 hours, while the expression of *SEC20* was repressed by doxycycline. Northern blot with 18S ribosomal RNA specific probe was used as a loading control. Bottom image: Western blot analysis of the His_6_-p33 level in the above yeast samples with anti-His antibody. (C) Reduced TBSV RNA production by the tombusvirus replicase assembled *in vitro* in cell-free extracts (CFEs) prepared from THC-SEC20 yeast strain grown in media supplemented with doxycycline. Purified recombinant p33 and p92^pol^ replication proteins of TBSV (from *E*. *coli*) and *in vitro* transcribed TBSV DI-72 (+)repRNA were added to the CFEs prepared from the shown yeast strains as shown schematically. Denaturing PAGE analysis shows the ^32^P-labeled TBSV repRNA products made by the reconstituted TBSV replicase. Each experiment was repeated. (D) Dsl1p is required for TBSV replication in yeast. Yeast strains RY261C and RY270D were generous gifts from Frederick M. Hughson (Princeton University). RY261C lacked wt *DSL1* and harbored plasmids pRS415 Dsl1L55E/L58D (Leu2 selection) and pRS416 (Ura3 selection). This mutation (L_55_E, dsl1m2) impairs Dsl1p and Tip20p interaction. Yeast strain RY270D lacked wt *DSL1* and harbored the plasmids pRS415 Dsl1A533D (Leu2 selection) and pRS416 (Ura3 selection). This mutation (A_533_D, dsl1m1) impairs Dsl1p and Sec39 interaction. The wt control strain harbored pRS415-DSL1 plasmid. Strains were transformed with HpGBK-CUP1-p33/ADH1-DI72 (His3 selection), then pRS416 plasmid was counter-selected on 5-fluoroorotic acid (5-FOA) plates at 23°C. Then, yeast strains were transformed with UpGBK-CUP1-p92. Transformed cells were grown in SC-ULH^-^ media supplemented with 2% glucose and 50 μM CuSO_4_ for 24 hours at 23°C, followed by total RNA extraction and Northern Blot analysis with a repRNA specific probe. Bottom image: Western blot analysis of the His_6_-p33 level in the above yeast samples with anti-His antibody. (E) Reduced TBSV RNA production by the tombusvirus replicase assembled *in vitro* in cell-free extracts (CFEs) prepared from dsl1m1 and above wt yeast strains. See further details in panel C. (F) Tip20p is required for TBSV replication in yeast. Yeast strain YAS2801 (TIP20::kanMX4 pRS315-tip20Δ1-81-LEU2) was a generous gift from Anne Spang (Biozentrum University of Basel, Switzerland). The yeast strain YAS2801 expresses Tip20p lacking the N terminal 81 amino acids, impairing its interaction with Dsl1p. The yeast strains YAS2801 and the corresponding wt SEY6210 were transformed with plasmids HpGBK-CUP1-6xHisp33/ADH1-DI72 and UpGBK-CUP1-p92. Transformed cells were grown in SC-ULH^-^ media supplemented with 2% glucose and 50 μM CuSO_4_ for 24 hours at 23°C, followed by total RNA extraction and Northern Blot analysis with a repRNA specific probe. Bottom image: Western blot analysis of the His_6_-p33 level in the above yeast samples with anti-His antibody.
(TIF)

S3 Fig Deletions of Sey1p and Yop1p proteins do not affect TBSV repRNA accumulation in yeast. (A-B) JHY4 (ΔSey1 ΔYop1: Sey1:: Kan-MX, Yop1::HIS3MX6), ACY 44 (Sey1:: hph) and the relevant wt strains were generous gifts from William Prinz, (NIH/NIDDK/LCBB). JHY4 and wt BY4741 were transformed with plasmids UpGBKADH1p33/GAL1-DI72 and LpGAD-CUP1p92 while ACY44 and the corresponding wt W303a strains were transformed with HpGBKCUP1p33/ADH1-DI72 and LpGADCUP1p92. RepRNA accumulation was induced with 2% galactose and 50 μM CuSO_4_ for 24 hours and then total RNA was extracted and Northern blot analysis was performed with a repRNA-specific 3’ end probe. (C) Different localization of Sey1p and p33 replication protein in wt yeast. The RFP-tagged Sey1p atlastin and the GFP-tagged p33 were detected by confocal microscopy. Top images show the most frequent and representative distribution of these two proteins, whereas the lower images show the infrequent proximal localization of RFP-Sey1p and GFP-p33. Strain SFNY 2134 that harbors chromosomally-tagged SEY1-5xRFP:: LEU2 was a generous gift from Susan Ferro-Novick (University of California, San Diego, HHMI). The strain was transformed with HispESC-GAL1-GFPp33. Cells were grown overnight in 2% glucose minimal media, then cells were washed and p33 expression was induced in 2% galactose media for 8 hours, followed by confocal laser imaging using Olympus FV1000 microscope.
(TIF)

S4 Fig Knockdown of *DSL1* gene inhibits TBSV RNA replication in *N*. *benthamiana* plants. Accumulation of the TBSV genomic (g)RNA in *DSL1* knockdown *N*. *benthamiana* plants 3 days post-inoculation, based on Northern blot analysis. Inoculation with TBSV gRNA was done 9 days after silencing of *DSL1* expression by sap inoculation. VIGS was performed via agroinfiltration of TRV vectors carrying *DSL1* sequence or the TRV-cGFP vector (as a control). Second panel: Ribosomal RNA is shown as a loading control. Note that the TBSV genomic RNA is also visible in the gel. Third panel: RT-PCR analysis of NbDsl1 mRNA level in the silenced and control plants. Fourth panel: RT-PCR analysis of *TUBULIN* mRNA level in the silenced and control plants. Each experiment was repeated.
(TIF)

S1 Text Experimental procedures. (DOCX)

S1 Table The names and sequences of oligo primers used in this study. (DOCX)
