# Supplementary figures and images for: Assembly-hub function of ER-localized SNARE proteins in biogenesis of tombusvirus replication compartment
Source: PLoS Pathog. 2018 May 10;14(5):e1007028. doi: 10.1371/journal.ppat.1007028 (PMC5963807; doi:10.1371/journal.ppat.1007028)

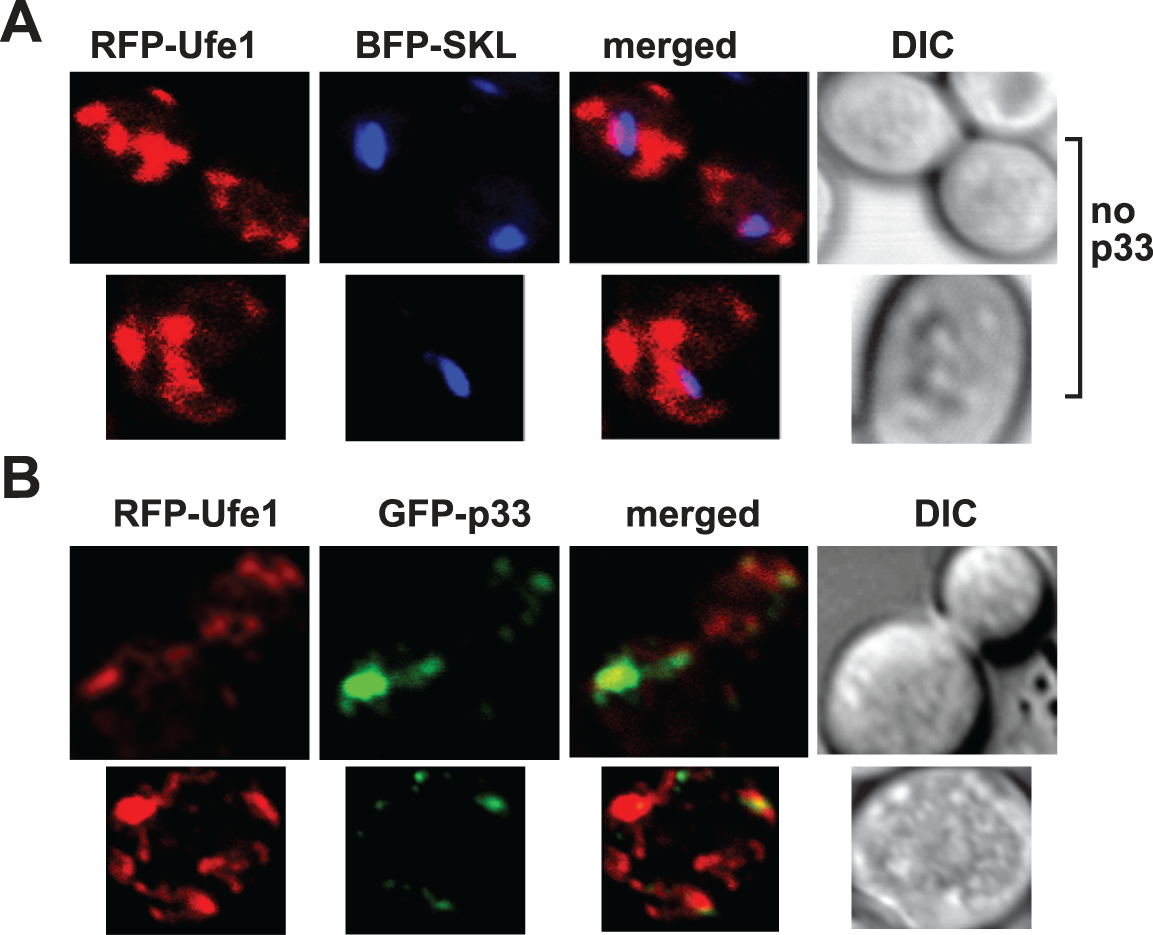

Supplement: S1 Fig — (A) Confocal microscopy images show the proximal localization of BFP-SKL (peroxisomal luminar marker) and RFP-Ufe1 ER SNARE protein in yeast lacking tombusviral components. (B) Confocal microscopy images show both proximal localization and co-localization of GFP-p33 and RFP-Ufe1 in wt yeast cells. (TIF) [file ppat.1007028.s001.tif]

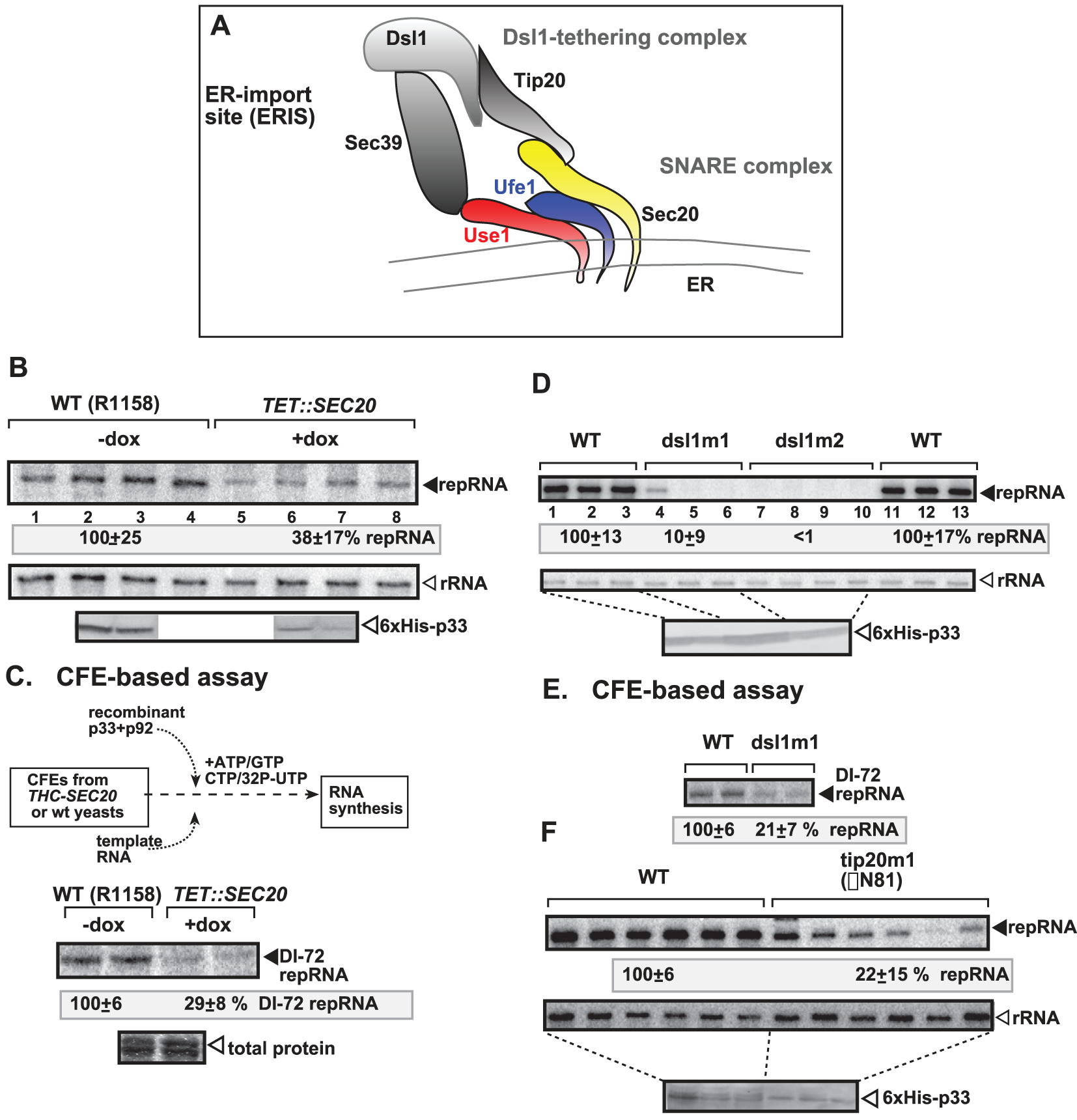

Supplement: S2 Fig — (A) Schematic representation of the ER SNARE complex (ERAS/ERIS subdomain of ER) with the bound dsl1-tethering complex containing three proteins. (B) Northern blot analysis of TBSV repRNA shows the reduced accumulation of repRNA in THC-SEC20 yeast strain. Viral proteins His6-p33 and His6-p92 were expressed from plasmids from the copper-inducible CUP1 promoter, whereas DI-72(+) repRNA was expressed from the galactose-inducible GAL1 promoter. TBSV replication was induced by growing yeast cells in media containing 2% galactose (also 2% raffinose as a carbone source) and 50 μM CuSO4 at 23°C for 36 hours, while the expression of SEC20 was repressed by doxycycline. Northern blot with 18S ribosomal RNA specific probe was used as a loading control. Bottom image: Western blot analysis of the His6-p33 level in the above yeast samples with anti-His antibody. (C) Reduced TBSV RNA production by the tombusvirus replicase assembled in vitro in cell-free extracts (CFEs) prepared from THC-SEC20 yeast strain grown in media supplemented with doxycycline. Purified recombinant p33 and p92pol replication proteins of TBSV (from E. coli) and in vitro transcribed TBSV DI-72 (+)repRNA were added to the CFEs prepared from the shown yeast strains as shown schematically. Denaturing PAGE analysis shows the 32P-labeled TBSV repRNA products made by the reconstituted TBSV replicase. Each experiment was repeated. (D) Dsl1p is required for TBSV replication in yeast. Yeast strains RY261C and RY270D were generous gifts from Frederick M. Hughson (Princeton University). RY261C lacked wt DSL1 and harbored plasmids pRS415 Dsl1L55E/L58D (Leu2 selection) and pRS416 (Ura3 selection). This mutation (L55E, dsl1m2) impairs Dsl1p and Tip20p interaction. Yeast strain RY270D lacked wt DSL1 and harbored the plasmids pRS415 Dsl1A533D (Leu2 selection) and pRS416 (Ura3 selection). This mutation (A533D, dsl1m1) impairs Dsl1p and Sec39 interaction. The wt control strain harbored pRS415-DSL1 plasmid. Strains were transfor [file ppat.1007028.s002.tif]

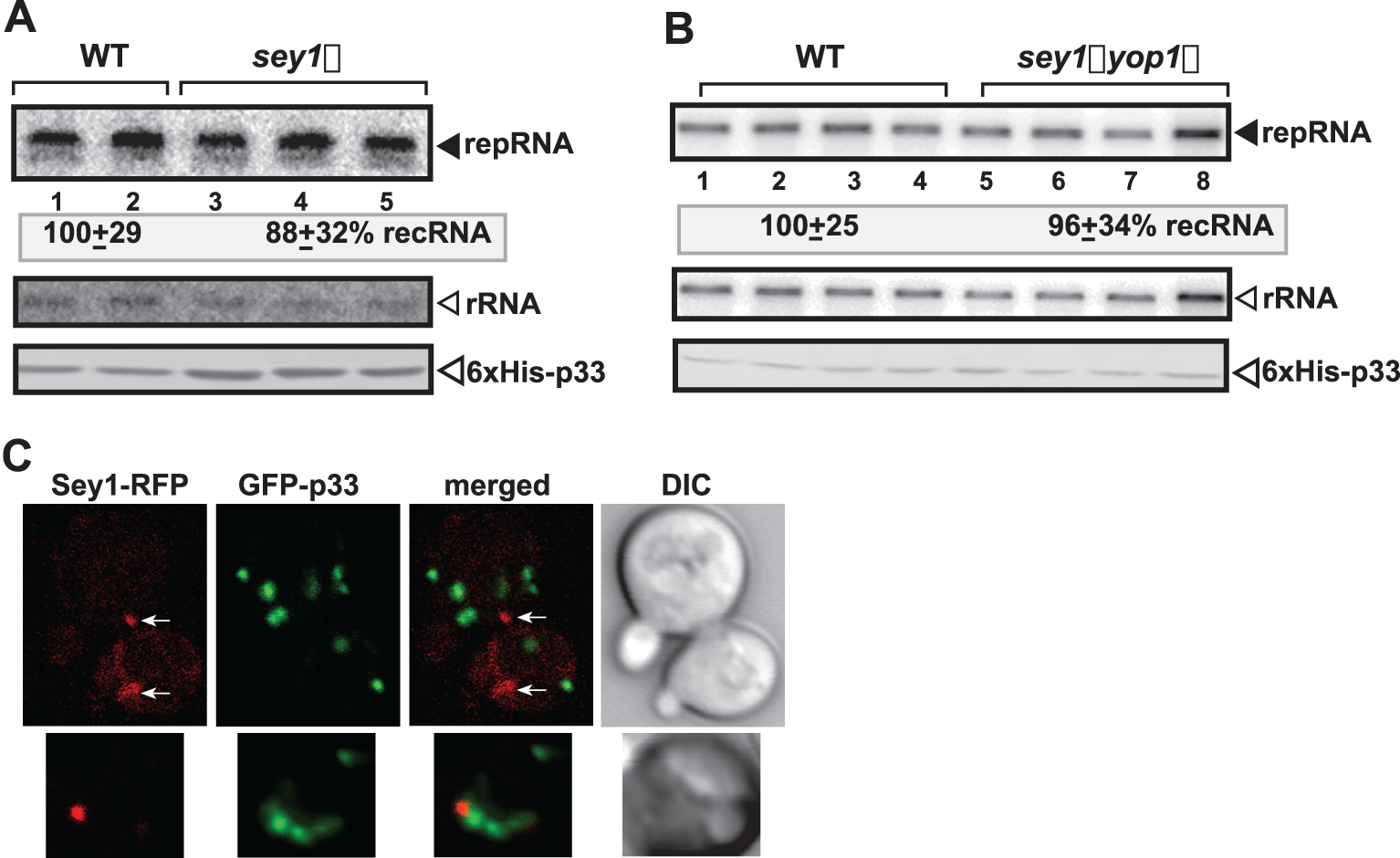

Supplement: S3 Fig — (A-B) JHY4 (ΔSey1 ΔYop1: Sey1:: Kan-MX, Yop1::HIS3MX6), ACY 44 (Sey1:: hph) and the relevant wt strains were generous gifts from William Prinz, (NIH/NIDDK/LCBB). JHY4 and wt BY4741 were transformed with plasmids UpGBKADH1p33/GAL1-DI72 and LpGAD-CUP1p92 while ACY44 and the corresponding wt W303a strains were transformed with HpGBKCUP1p33/ADH1-DI72 and LpGADCUP1p92. RepRNA accumulation was induced with 2% galactose and 50 μM CuSO4 for 24 hours and then total RNA was extracted and Northern blot analysis was performed with a repRNA-specific 3’ end probe. (C) Different localization of Sey1p and p33 replication protein in wt yeast. The RFP-tagged Sey1p atlastin and the GFP-tagged p33 were detected by confocal microscopy. Top images show the most frequent and representative distribution of these two proteins, whereas the lower images show the infrequent proximal localization of RFP-Sey1p and GFP-p33. Strain SFNY 2134 that harbors chromosomally-tagged SEY1-5xRFP:: LEU2 was a generous gift from Susan Ferro-Novick (University of California, San Diego, HHMI). The strain was transformed with HispESC-GAL1-GFPp33. Cells were grown overnight in 2% glucose minimal media, then cells were washed and p33 expression was induced in 2% galactose media for 8 hours, followed by confocal laser imaging using Olympus FV1000 microscope. (TIF) [file ppat.1007028.s003.tif]

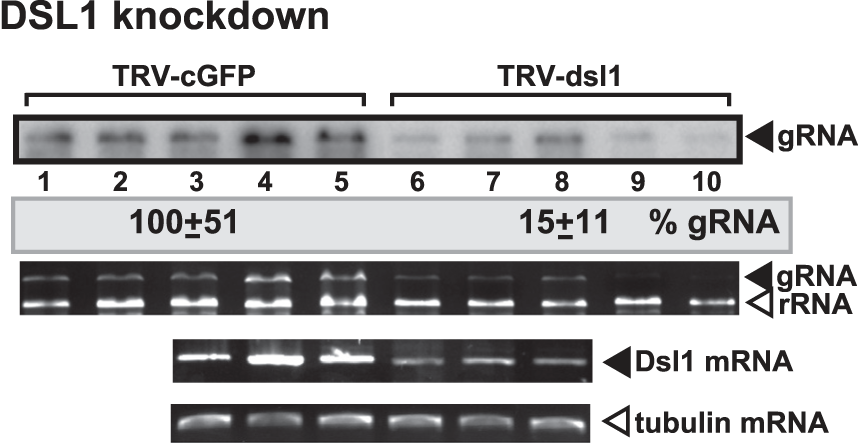

Supplement: S4 Fig — Accumulation of the TBSV genomic (g)RNA in DSL1 knockdown N. benthamiana plants 3 days post-inoculation, based on Northern blot analysis. Inoculation with TBSV gRNA was done 9 days after silencing of DSL1 expression by sap inoculation. VIGS was performed via agroinfiltration of TRV vectors carrying DSL1 sequence or the TRV-cGFP vector (as a control). Second panel: Ribosomal RNA is shown as a loading control. Note that the TBSV genomic RNA is also visible in the gel. Third panel: RT-PCR analysis of NbDsl1 mRNA level in the silenced and control plants. Fourth panel: RT-PCR analysis of TUBULIN mRNA level in the silenced and control plants. Each experiment was repeated. (TIF) [file ppat.1007028.s004.tif]
