# Supplementary material for: Assembly-hub function of ER-localized SNARE proteins in biogenesis of tombusvirus replication compartment
Source: PLoS Pathog. 2018 May 10;14(5):e1007028. doi: 10.1371/journal.ppat.1007028 (PMC5963807; doi:10.1371/journal.ppat.1007028)
Supplement: S1 Text — (DOCX) [file ppat.1007028.s005.docx]

**S1 Text. Experimental Procedures**

**Yeast trains and expression plasmids**

*Sacchromyces cerevisiae* strain BY4741 (*MATa* *his3Δ1 leu2Δ0 met15Δ0 ura3Δ0*), ΔPEX19 (BY4741 PEX19:: kanMX), THC-SEC39, tetO7:: SEC20-G418 and R1158 (wt) strains [1] were obtained from Open Biosystems. Yeast strains RY261C and RY270D were generous gifts from Dr. Frederick M. Hughson (Princeton University). Yeast strains YAS2801 (TIP20::kanMX4 pRS315-tip20Δ1-81-LEU2) and the corresponding WT SEY6210 were a generous gift from Dr. Anne Spang (Biozentrum, University of Basel, Switzerland) [2]. Yeast strains JHY4 (ΔSey1 ΔYop1: Sey1:: Kan-MX, Yop1::HIS3MX6), ACY 44 (Sey1:: hph) and the relevant WT strains were generous gifts from Dr. William Prinz, (NIH/NIDDK/LCBB) [3,4]. Yeast strain SFNY 2134 that harbored chromosomally tagged SEY1-5xRFP:: LEU2 was a generous gift from Dr. Susan Ferro-Novick (University of California, San Diego, HHMI) [5].

BY4741-based yeast strain UFE1::GALS-3xHA-Ufe1-Nat (referenced as GALS::UFE1 in the text) and USE1::GAL1-3xHA-Use1-Nat (referenced as GAL1::USE1 in the text) yeast strains expressing 3xHA-tagged Ufe1 under *GAL1S* promoter (the short version of *GAL1* promoter) and 3xHA-tagged Use1 under *GAL1* promoter, respectively, were created by using plasmid pYM-N32 and pYM-N24 from the EUROSCARF plasmid collection according to authors’ recommendation [6]. BY4741-based yeast strain OSH6::GALS-3xHA-Osh6-natNT2 expressing *GAL1* promoter-driven 3xHA-tagged Osh6 protein was created by using plasmid pYM-N32 from the EUROSCARF plasmid collection.

Yeast strain with Tet promoter controlled *COP1* expression from genome was from Tet-promoters Hughes Collection (yTHC) obtained from Open Biosystems [1]. *URA3* gene, which marks the tet activator from Tet-COP1 strain was then removed by homologous recombination. Briefly, a DNA fragment containing URA3 5'-terminal and 3'-terminal sequences was PCR-amplified from pFA6-hphNT1 (euroscarf) using primer pair #5483 / #5484, and then transformed into Tet-COP1 yeast strain. Resulting *URA3* deleted Tet-COP1 yeast strain was selected using hygromycin B selection, thus enabling the use of plasmids containing *URA3* selection.

The resulting Tet-COP1 yeast strain (*ura3*Δ) was transformed with pESC-T33/DI72 and pYES-T92[7] together with pRS315 or pRS315-COP1. Yeast transformants were grown in synthetic complete medium lacking uracil, histidine, leucine (SC-ULH^-^) and containing 2% glucose overnight at 29 °C, then cultured in synthetic complete medium lacking uracil, histidine, leucine, supplemented with 2% glucose with or without addition of 10 μg/ml doxycycline at a starting OD_600_ of 0.2 for 6 hours at 29 °C, then washed into synthetic complete medium lacking uracil, histidine, leucine and containing 2% galactose medium with or without 10 μg/ml doxycycline at a starting OD_600_ of 1.0, and cultured for 24 hours at 23 °C to induce viral replication. Yeast cell cultures were harvested and subjected to RNA extraction and Northern hybridization analysis.

To construct pGAD-BT2-N-HisUfe1, *UFE1* sequence was PCR-amplified with primers #5774/#5775 that included restriction sites SpeI and NcoI. The obtained PCR product was digested with SpeI and NcoI and then, ligated into pGAD-BT2-N digested with the same set of restriction enzymes. To create pPR-N-RE plasmids the following primer pairs and restriction enzymes were used: pPR-N-RE-Ufe1 (primers used: #5362BamHI/#5590XhoI), pPR-N-RE-Syp81 (primers used: #5880BamHI/#5881XhoI), pPR-N-RE-Use1 (primers used: #5364/#5365), pPR-N-RE-Dsl1 (primers used: #5224BglII/#5225NheI), pPR-N-RE- Sec20 (primers used: #5220BglII/#5221NheI), pPR-N-RE-Erg9 (primers used: #5871BamHI/#5971NheI), pPR-N-RE-p36 (primers used: #2632BamHI/#3665XhoI). Before ligation, pPR-N-RE vectors were digested with the corresponding enzymes with the exception that SalI was used instead of XhoI.

To create plasmid LpGAD-ADH1-RFP-6xHis-ScUfe1, full-length *UFE1* sequence was PCR-amplified using purified genomic DNA of BY4741 as a template with primer pair #5362/#5590, then the obtained PCR product was digested with BamHI and XhoI restriction enzymes. The digested PCR product was ligated into LpGAD-ADH1-RFP-6xHis plasmid [8] digested with the same restriction enzymes. Plasmid LpGAD-ADH1-RFP-6xHis-ERG9 was created in a similar way, the sequence of full-length *ERG9* was PCR-amplified with primers #5871/#5872. UpYES-GAL1-GFP-p33 was created previously [9].

Plasmid UpYC-GAL1-Ufe1ΔTM was created to express the untagged Ufe1ΔTM. The last 34 amino acids at the C-terminus containing the trans-membrane region were deleted in Ufe1ΔTM [10]. Partial Ufe1 sequence was PCR-amplified on the genomic DNA purified from BY4741 as a template with the primer pair #5362 and #5398. Then the PCR product was digested with BamHI and XhoI restriction enzymes and ligated into pYC low copy number plasmid digested with the same restriction enzymes.

Plasmid pGD-35S-YFP-Syp81 was created by RT-PCR-amplification of *Arabidopsis thaliana* *SYP81* (AT1G51740) sequence with primers #5880/#5881, then the obtained RT-PCR product was digested with BamHI. YFP sequence was PCR-amplified with primers #1941/#1295 and plasmid pYES2/NTC-YFP-p33 used as a template [9]. The PCR product of YFP was digested with BamHI, and then ligated with the digested *SYP81* PCR product. Then the ligated product was PCR-amplified with primers #1941/#5881 and digested with BglII and SalI. The digested fragment was ligated to the pGD-35S plasmid digested with the same restriction enzymes. Plasmid pGD-35S-RFP-Syp81 was created in a similar way. To create pGD-35S-YFP-*At*Use1 the sequence of *AtUSE1* (AT1G54110) was PCR-amplified with primers #5364/#6097. The obtained PCR fragment was digested with BamHI and ligated together with the PCR-amplified YFP digested with BamHI. Then the ligated fragments were PCR-amplified, then digested with BglII and XhoI and cloned into the plasmid digested with the same restriction enzymes. pGD-35S-YFP-SQS plasmid was created as follows. *SQS1* (At4g34640) is the plant homologue of *ScERG9* [11]. Full-length *SQS1* sequence was RT-PCR-amplified with primers #6076/#6077. YFP sequence was PCR-amplified with primers #5905/#6056. Both PCR products were digested with BamHI then ligated together. YFP-SQS1 sequence was PCR-amplified with primers #5905/#6077, then digested with BglII and XhoI and ligated to pGD vector digested with the same restriction enzymes. pGD-35S-p33-BFP, pGD-35S-p33-GFP and  pGD-35S-BFP_SKL_ plasmids were created as described earlier [12].

To create plasmid pGD-35S-cYFP-Syp81, C-terminal part of YFP sequence was PCR amplified with primers #5908/#6056 and *At*Syp81 full-length sequence was PCR-amplified with primers #5880/ #5881. And, then both PCR products were digested with BamHI restriction enzyme. After ligation, PCR-amplification was performed with primers #5908 and #5881, digested with BglII and XhoI and ligated to plasmid pGD-35S digested with the same restriction enzymes. pGD-35S-nYFP-*At*Use1 was created in a similar way. The N-terminal fragment of YFP was PCR-amplified with primers #5905/#6069 and *At*Use1 was PCR-amplified with primers #5298/ #6055. The fragments were digested with BamHI and BglII respectively, then, they were ligated together. Then, the ligation product was PCR-amplified with primers #5905 and #6055, digested with BglII and XhoI and ligated to plasmid pGD-35S digested with the same restriction enzymes.

Plasmid pGD-35S-Syp81ΔTM was created by PCR-amplification of the sequence of Syp81 with primers #5880/ #5533 and then digested with BamHI and SalI. The fragment was ligated into pGD-35S plasmid digested with the same restriction enzymes.

To generate pTef1 promoter driven *COP1* (without a tag) expression plasmid, PCR fragment of *COP1* was amplified from yeast genome using primer pair #1858/ #1859, digested with BamHI/XhoI and inserted into BamHI/SalI digested pRS315-pTef1 [13], generating pRS315-COP1. C-terminally Flag-tagged ARF1 mutants were generated via site directed mutagenesis and then PCR-amplified with primer pair #3132/ #5742, digested with BamHI/PstI, and inserted into pRS315-pCUP1-CFlag [14].

To obtain plasmids UpESC-Cup-His-Osh6 and UpESC-Cup-His-AtVap27-1, first, Osh6 and AtVap27-1 sequences were PCR-amplified using pYC(Ura)-Gal-HisOsh6, pYES(Ura)-Gal-HisAtVap27-1 [12] as a templates and primers #7597 (CCAGGGATCCATGGGTCATCATCATCATCATCATATGGGCTCCAAAAAACTGACCG) and #5132 (CGGCTCGAGGCTAGCCTATTGTTTTGCTGGGTTCTG), or #7596 (CCAGGGATCCATGGGTCATCATCATCATCATCATATGAGTAACA-TCGATCTGATTGGG) and #3459 (CGGCTCGAGTTAGCTAGCTGTCCTCTTCATAATGTATCC) for Osh6 and AtVap27-1, respectively. The obtained PCR products were digested with BamHI and XhoI restriction enzymes and ligated to the UpESC-Cup-notag plasmid, digested with BamHI and XhoI.

**Analysis of *in vivo* protein interactions by split-ubiquitin assay in yeast**

Yeast membrane two-hybrid (split-ubiquitin) assay (Dualsystems), was performed to analyze p33 interaction with the SNARE and tethering proteins. Experiment was performed as described earlier [15]. The plasmids pGAD-BT2-N-Hisp33 [8] or pGAD-BT2-N-HisUfe1 were co-transformed with pPR-N-RE derived constructs into the reporter yeast strain NMY51. The transformed yeast colonies were re-suspended in 100 µl of water and serially diluted (10-fold) in water. 5 µl of each dilution were spotted onto TLHA^-^ plates to score for interaction, or onto TL^-^ plates, as growth controls. To explore the effect of the presence of p33 on the interactions of Ufe1 with Ufe1 and Erg9, the plasmids UpGBK-ADH1-His-p33/GAL1-DI72 or UpYC empty were co-transformed with the relevant pGAD-BT2-N and pPR-N-RE plasmids and UTLHA^-^ or UTL^-^ plates were used.

**Recombinant protein purification from *E. coli***

For the expression and purification of the recombinant MBP-tagged TBSV p33 and p92 replication proteins from *E. coli,* we followed a published protocol [16]. Briefly, the expression plasmids were transformed into *E. coli* strain BL21(DE3) CodonPlus. Isopropyl β-D-thiogalactopyranoside (IPTG) was used for inducing protein expression for 8 h at 16°C. After the cells were collected by centrifugation (5,000 rpm for 5 min), they were suspended and sonicated in MBP column buffer (30 mM HEPES-KOH pH 7.4, 25 mM NaCl, 1 mM EDTA, 10 mM β-mercaptoethanol). The extract was centrifuged at 14,000 rpm for 5 min to remove cell debris and then supernatant was incubated with amylose resin (NEB) for 15 min at 4°C with mixing. The resin was washed 5 times with the column buffer and the bound proteins were eluted with column buffer containing 0.18% (W/V) maltose.

Eluted proteins were aliquoted and stored at -80°C. Proteins used for the replication assays were at least 95% pure, as determined by SDS-PAGE (not shown).

***In vitro* TBSV replication assay in cell-free yeast extract**

Yeast strains BY4741, UFE:Gal1S, and USE:Gal1S were pre-grown for 16 h at 29°C in YPG media supplemented with 2% raffinose. After yeast cells were centrifuged at 3,000 rpm for 5 min and washed with YPD media, yeasts were grown for 8 h at 29°C in YPD media. We followed a previously published protocol [17] for the preparation of cell-free yeast extracts capable of supporting TBSV replication *in vitro*. The *in vitro* TBSV replication assays were performed in 20 µl total volume containing 3 µl of CFE, 0.25 µg DI-72 (+)repRNA transcript, 200 ng purified MBP-p33, 200 ng purified MBP-p92^pol^ (both recombinant proteins were purified from *E. coli*), 30 mM HEPES-KOH, pH 7.4, 150 mM potassium acetate, 5 mM magnesium acetate, 0.13 M sorbitol, 0.4 µl actinomycin D (5 mg/ml), 2 µl of 150 mM creatine phosphate, 0.2 µl of 10 mg/ml creatine kinase, 0.2 µl of RNase inhibitor, 2 µl of 0.1 M dithiothreitol (DTT), 2 µl of rNTP mixture (10 mM ATP, CTP, and GTP and 0.25 mM UTP) and 0.1 µl of [^32^P]UTP [17]. After incubation at 25°C for 3 h, the RNA products synthesized in the replication assays were separated by electrophoresis in 0.5x Tris-borate-EDTA buffer in a 5% polyacrylamide gel containing 8 M urea.

**Detection of PE distribution based on confocal laser microscopy**

Distribution of PE was studied in Ufe1 and Use1 knock-down yeast strains. Briefly, BY4741-based UFE1::GALS-3xHA-Use1-Nat and BY4741-based USE1::GAL1-3xHA-Use1-Nat yeast strains were transformed with pRS425-pCUP1-GFP-p33 [18] or with pRS425 empty plasmid as a control. Cells were grown in SC-L^-^ media supplemented with 2% raffinose and 0.5% galactose in the presence of 100 µM bathocuproine disulphonate (BCS) overnight, then medium was changed to SC-L^-^ media supplemented 2% raffinose and 0.5% glucose for 6 h to inhibit the expression of Ufe1 or Use1. Then, BCS was washed out and fresh SC-L^-^ media supplemented with 2% raffinose and 0.5% glucose containing 50 μM CuSO_4_ was provided to induce GFP-p33 expression. After 16 h, yeast cells were harvested and PE distribution was visualized as described earlier [19], except that the PE probe biotinylated-duramycin was detected by Streptavidin conjugated with Alexa Fluor 594 (Life Technologies), showing red color under confocal microscopy.

**Co-localization assays in planta**

For the co-localization assay, plasmids pGD-35S-YFP-Syp81, pGD-35S-RFP-Syp81, pGD-35S-YFP-*At*Use1, pGD-35S-YFP-SQS1, pGD-35S-p33-BFP, pGD-35S-p33-GFP and  pGD-35S-BFP_SKL_ were transformed into Agrobacterium strain C58C1Rif. Four weeks old N. benthamiana leaves were agroinfiltrated for the transient expression of the tagged proteins (according to figure labels). Agroinfiltrated leaves were analyzed 48 h after agro-infiltration and confocal laser imaging was performed using Olympus FV1000 laser scanning microscope [20]. Co-localization of RFP-Sey1 and p33 replication protein was tested in yeast strain SFNY 2134, which harbored a chromosomally tagged SEY1-5xRFP:: LEU2 [5]. The yeast strain was transformed with HispESC-GAL1-GFPp33. Yeast cells were grown overnight in minimal medium supplemented with 2% glucose, then yeast cells were washed and p33 expression was induced in minimal medium supplemented with 2% galactose for 8 h. Then, confocal laser imaging was performed using Olympus FV1000 microscope.

**Bimolecular Fluorescence Complementation Assay in planta**

Plasmids pGD-35S-cYFP-Syp81 and pGD-35S-nYFP-*At*Use1 were co-transformed along with pGD-35S-p33-BFP into Agrobacterium strain C58C1Rif. Four weeks old N. benthamiana leaves were agroinfiltrated (OD_600_ value of 0.3) for the transient expression of the tagged proteins. Confocal images were taken 3 days after agroinfiltration. The fluorescence complementation was detected via the GFP channel (excitation/emission: 488 nm/500–530 nm) and BFP was detected at excitation 405 nm [12].

**TBSV replication assays in yeast**

Assay #1 was performed as follows: WT BY4741 and ΔPEX19 strains were transformed with plasmids HpGBK-CUP1-p33/ADH1-Di72, UpESC-CUP1-p92 and LpGAD-ADH1-RFP-6xHIS-Ufe1ΔTM or empty LpGAD plasmid as a control. Transformed yeast strains were grown in minimal media supplemented with 2% glucose. Ufe1ΔTM was expressed under *ADH1* constitutive promoter and TBSV RNA replication was induced with 50 μM CuSO_4_. Yeast cells were harvested after 24 h. Then, total RNA was extracted and Northern blot analysis performed with 3′ specific P^32^-labeled probe to the TBSV repRNA [21]. Expressed proteins were detected with anti-His antibody, followed by alkaline phosphatase-conjugated anti-mouse antibody and NBT-BCIP detection.

Assay #2 was performed as follows: BY4741 strain was transformed with plasmids HpGBK-CUP1-p33/ADH1-DI72, LpGAD-CUP1-p92 and UpYC-GAL1-Ufe1ΔTM or UpYC empty plasmid as a control. Transformed yeast strains were grown in 2 ml minimal medium supplemented with 2% glucose in the presence of 100 µM bathocuproine disulphonate (BCS) copper chelator for 6 h and then yeast cells were washed and minimal medium supplemented with 2% galactose was added to induce Ufe1-ΔTM expression. After overnight growth at 23°C, BCS was removed by changing to fresh minimal medium supplemented with 2% galactose and TBSV repRNA replication was induced with 50 μM CuSO_4_. After 24 h, yeast cells were harvested, total RNA was extracted and Northern blot analysis was performed.

In another set of experiment (when virus replication was induced before the expression of Ufe1-ΔTM), transformed yeast strains were grown in 2 ml minimal medium supplemented with 2% glucose in the presence of 50 μM CuSO_4_ for 6 h and then yeast cells were washed and the media was changed to minimal medium supplemented with 2% galactose containing 50 μM CuSO_4_ to induce Ufe1-ΔTM expression. Yeast cells were harvested after 24 h of TBSV replication. Then total RNA was extracted and Northern blot analysis was performed.

**TBSV repRNA replication in Sec20 depleted yeast strain**

WT R1158 and tetO7:: SEC20-G418 yeast strains were transformed with pGBK-CUP1-6xHisp33/ADH1-DI-72 (his3 selection) together with pGAD-CUP1-6xHisp92 (leu2 selection). Yeast cells were grown overnight in SC-LH^−^  supplemented with 2% glucose at 23 °C in the presence of 100 µM BCS. Then, to down-regulate the expression of Sec20, doxycycline (20 µg/ml) was added to the media for 9 h. Then, yeast cells were centrifuged and washed thoroughly, followed by culturing in SC-LH^−^ glucose media containing 20 µg/ml doxycycline and 50 µM CuSO_4_ to induce viral protein expression. Yeast cells were grown at 23 °C for 24 h. Then, total RNA was extracted, phenol/chloroform precipitated and the TBSV repRNA accumulation was detected by Northern blot analysis with 3′ specific P^32^-labeled probe to the repRNA.

**TBSV repRNA** **replication in dsl1 mutant yeast strains**

Yeast strain RY261C lacked wt *DSL1* and harbored the plasmids pRS415 Dsl1L55E/L58D (Leu2 selection) and pRS416 (Ura3 selection). This mutation impairs Dsl1 and Tip20 interaction (dsl1m2). RY270D lacked wt DSL1 and harbored the plasmids pRS415 Dsl1A533D (Leu2 selection) and pRS416 (Ura3 selection). This mutation impairs Dsl1 and Sec39 interaction (dsl1m1) [22]. The wt control strain harbored pRS415-DSL1 plasmid. Strains were transformed with HpGBK-CUP1-p33/ADH1-DI72 (His3 selection) then pRS416 plasmid was counter-selected on 5-fluoroorotic acid (5-FOA) plates at 23°C. Then, yeast strains were transformed with UpGBK-CUP1-p92. Transformed yeast cells were grown in SC-ULH^-^ media containing 2% glucose and 50 μM CuSO_4_ for 24 h at 23°C and then total RNA was extracted and Northern Blot analysis was performed with a repRNA specific probe.

**TBSV repRNA** **replication in tip20 mutant yeast strain**

Yeast strains YAS2801 (mutant Tip20 lacking the N-terminal 81 amino acids, thus interaction with Dsl1 is impaired [23] and the corresponding WT yeast strain SEY6210 were transformed with plasmids HpGBK-CUP1-6xHisp33/ADH1-DI72 and UpGBK-CUP1-p92. Yeast cells were grown in SC-ULH^-^ media supplemented with 2% glucose and 50 μM CuSO_4_ for 24 h at 23 ºC and then total RNA was extracted and Northern Blot analysis was performed with a repRNA specific probe. Expression of His_6_-tagged p33 was detected with anti-His antibody.

**Effect of the deletion of Sey1 and double deletion** Δ**Sey1**Δ**Yop1 on TBSV repRNA accumulation**

JHY4 [5] and WT BY4741 yeast strains were transformed with plasmids UpGBKADH1p33/GAL1-DI72 and LpGAD-CUP1p92, whereas yeast strains ACY44 and the corresponding WT W303a strains were transformed with HpGBKCUP1p33/ADH1-DI72 and LpGADCUP1p92. TBSV repRNA accumulation in yeasts was induced in SC-ULH^-^ media supplemented with 2% galactose and 50 μM CuSO_4_ for 24 h and then total RNA was extracted and Northern Blot analysis was performed with a repRNA specific probe.

**Co-purification of Ufe1 or Use1 with the viral replicase in yeast**

For co-purification of the yeast Ufe1 and Use1 proteins with the membrane-bound p33 and p92 replication proteins, yeast strains BY4741-based UFE1::GALS-3xHA-Ufe1-natNT2 and the BY4741-based UFE1::GAL1-3xHA-Use1-natNT2 were co-transformed with plasmids HpGBK-CUP1-FLAGp33/GAL1-DI-72 and LpGAD-CUP1-Hisp92 (or HpGBK-CUP1-Hisp33/Gal1-DI-72 and LpGAD-CUP1-Hisp92 as a control). Transformed yeasts were pre-grown in selective SC-LH^−^ medium supplemented with 1% raffinose and 1% galactose plus BCS for 24 h at 23°C and then transferred to selective medium supplemented with 1% raffinose and 1% galactose with 50 μM CuSO_4_ to induce expression of FLAG-p33 and His_6_-p92 or His_6_-p33 from the CUP1 promoter, and yeast cultures were grown for an additional 6 h at 23°C. Then, the cultures were centrifuged, washed once with phosphate-buffered saline (PBS), and then incubated in PBS buffer containing 1% formaldehyde for 1 h on ice to cross-link proteins [24]. Formaldehyde was quenched by addition of glycine (0.1M final concentration). Finally, yeast cultures were washed in PBS and the viral replicase was FLAG-affinity purified using anti-FLAG M2 agarose as described [25]. Purified FLAG-p33 was analyzed by Western blotting with anti-FLAG antibody, followed by anti-mouse antibody conjugated to alkaline phosphatase. His-tagged p33 was analyzed with anti-His-antibody followed by anti-mouse antibody conjugated to alkaline phosphatase. Co-purified HA-tagged host proteins were analyzed with anti-HA antibody, followed by alkaline phosphatase-conjugated anti-rabbit antibody and detection with NBT-BCIP as described previously as described previously [12].

**Co-purification of p33 and Osh6 in the presence of Ufe1-ΔTM in yeast**

For co-purification of the yeast Osh6p protein with the membrane-bound viral replicase (p33 and p92 replication proteins), yeast strains BY4741-based OSH6::GALS-3xHA-Osh6-natNT2 was co-transformed with plasmids HpGBK-CUP1-FLAGp33/GAL1-DI-72, LpGAD-CUP1-Hisp92 and UpGBK-ADH1-6xHisUfe1ΔTM (or HpGBK-CUP1-Flagp33/Gal1-DI-72, LpGAD-CUP1-Hisp92 and empty vector with Ura3 selection as a control). Transformed yeasts were pre-grown in selective SC-ULH^−^ medium supplemented with 2% glucose + BCS for 12 h at 23°C and then transferred to selective medium supplemented with 2% galactose for 24 h at 23°C to induce 3xHA-Osh6 protein expression from the GAL1 promoter. Then the cultures were supplemented with 50 μM CuSO_4_ to induce expression of FLAG-p33 and His_6_-p92 or His_6_-p33 and His_6_-p92 from the CUP1 promoter, and yeast cultures were grown for an additional 4 h at 23°C. The membrane-bound viral replicase was purified and the amounts of FLAG-p33 and 3xHA-Osh6 were determined as described above.

**Co-purification of the viral replicase from Gal::UFE1 and Gal::USE1 yeasts**

Yeast strains BY4741, Gal::UFE1 and Gal::USE1 were transformed with the following plasmids pGBK-HIS-Cup-Flag33/Gal-DI-72 [26] expressing Flag-tagged p33 of cucumber necrosis virus (CNV) and the TBSV DI-72 repRNA, pGAD-LEU- Cup-Flag92 expressing Flag-tagged p92 of CNV and one of two plasmids UpESC-Cup-His-Osh6 or UpESC-Cup-His-AtVap27-1, expressing His-tagged Osh6 and AtVap27-1 host factors, respectively. Transformed yeasts were grown for 18 h in SC-ULH^−^ media supplemented with 2% galactose and 100 μM BCS at 29°C. After yeast cells were centrifuged at 2,000 rpm for 3 min and washed with SC-ULH^−^ medium supplemented with 2% glucose, yeast pellet was resuspended in SC-ULH^−^ medium supplemented with 2% glucose and 100 μM BCS. After growing yeasts for 24 h at 29°C, the medium was changed to the ULH^−^ medium containing 2% glucose and 50 µM CuSO_4_ and culturing continued for 10 h at 23°C. Yeasts were pelleted and the Flag-tagged replicase was purified following previously published protocol [27]. Balancing for total fraction after breaking cells and debris removal was based on total proteins amount, while balancing a fraction, eluted from anti-FLAG M2-agarose affinity resin column was based on purified Flag-p33 amount. Both total and purified fractions were analyzed on the presence of His-tagged host proteins. Purified Flag-p33 was detected by western blot using anti-Flag antibody, co-purified His-tagged host proteins were detected with anti-His antibody, followed by anti-mouse antibody conjugated to alkaline phosphatase. Colorimetric detection was performed with NBT and BCIP.

All experiments have been repeated at least three times. Quantification and comparison of the amounts of proteins co-purified with Flag-tagged replicase was done as follows. First, total protein levels in protein samples obtained from WT and mutant yeasts were adjusted (same amount of total proteins were loaded) based on the Coomassie Brilliant Blue stained SDS-PAGE gel. The levels of expression of 6xHis-tagged host factors in adjusted samples from WT and mutant yeast strains were compared after scanning the Western blot (anti-His antibody) and its quantification with ImageQuant software. Next, proteins obtained after Flag-purification from WT and mutant yeasts were adjusted using Flag-p33 levels (the same amount of p33 protein were loaded from each preparation) based on the SDS-PAGE gel followed by Western blot with anti-Flag antibody. The levels of co-purified 6xHis-tagged host factors in adjusted Flag-purified samples from WT and mutant yeast strains were compared after scanning the Western blot (anti-His antibody) and its quantification. Normalization of amounts of co-purified 6xHis-tagged host factors has been done in case of the difference in the expression levels of 6xHis-tagged host factors in total samples have been detected. Finally, adjusted amounts of co-purified 6xHis-tagged host factors from mutant yeast strains were divided by adjusted amounts of co-purified 6xHis-tagged host factors from WT yeast strain (taken as 100%).

**Filipin-based staining of ergosterol distribution in the presence and absence of Ufe1-ΔTM**

To examine the distribution of ergosterol during TBSV replication in yeast in the presence and absence of Ufe1-ΔTM, BY4741 yeast strain was co-transformed with plasmids HispGBK-CUP1-Hisp33/Gal1-DI-72 and UpESC-CUP1-Hisp92 and LEUpGAD-ADH1-HisRFPUfe1-ΔTM. Control untransformed and transformed yeasts were grown in SC minimal media supplemented with 2% galactose and 50 μM CuSO_4_ for 24 hours at 23°C. Cultures were fixed with 3% formaldehyde for 1 h at room temperature. Formaldehyde was quenched by addition of glycine (0.1M final concentration). Fixed cells were centrifuged and washed twice with distilled water. Washed cells were incubated with 5 mg/ml filipin complex (Sigma Chemicals) in the dark for 15 min at 23°. Filipin-based fluorescence was observed by spotting 2-3 μl of the cell suspensions onto poly-L-lysine coated microscope slides under UV light microscope using DAPI filter [12].

**Silencing of *DSL1* in *N. benthamiana***

*Saccharomyces* *DSL1* is a homolog of mammalian ZW10 [28,29]. And the mammalian ZW10 is a putative homolog of *Arabidopsis* ZW10 like protein, AT2G32900.1. (see TAIR and NCBI databases and ref. [30]. To find the relevant tobacco sequence, full-length AT2G32900.1 sequence was blast-searched for tomato sequences in Gene Indices database. Then the highest score hit nucleotide sequence, LeBF051312 was blast-searched against *Nicotiana tabacum* database (Gene Indices, Harvard University). The best identity of 88% was found with FG141346 TC sequence. The relevant *Solanum lycopersicum* sequence ID: XM_004240106.2 and the relevant *Nicotiana tabacum* sequence XM_016586047.1 can be found in the NCBI database. To create the VIGS vector (pTRV2-*Nt*ZW10like), a 245-bp cDNA fragment was RT-PCR-amplified from a total RNA extract of *N. benthamiana* leaves using the primer pairs: #5588 and #5589 harboring restriction sites BamHI and XhoI, respectively. After digestion, the PCR product was ligated to the pTRV2 plasmid digested with BamHI and XhoI. Agrobacterium cultures transformed with either plasmid pTRV2-*Nt*ZW10like or pTRV2-cGFP as control and then were agroinfitrated (OD_600_ value 0.5) into *N. benthamiana* plants. On the 8^th^ day of agroinfiltration, leaves were sap inoculated with TBSV or TMV. Then, total RNA was extracted on the 2^nd^ day post-inoculation from the infected leaves, followed by Northern blot analysis. Silencing of the target gene was confirmed with primers #5588/#5648 on total RNA extract of pTRV2-NtZW10like and pTRV2-cGFP agroinfiltrated plants. In both experiments (SYP81 and ZW10 silenced plants) RT-PCR amplification of tubulin mRNA sequence with primers F2860/R2859 was performed as control.

**References:**

1. Mnaimneh S, Davierwala AP, Haynes J, Moffat J, Peng WT, et al. (2004) Exploration of essential gene functions via titratable promoter alleles. Cell 118: 31-44.

2. Diefenbacher M, Thorsteinsdottir H, Spang A (2011) The Dsl1 tethering complex actively participates in soluble NSF (N-ethylmaleimide-sensitive factor) attachment protein receptor (SNARE) complex assembly at the endoplasmic reticulum in Saccharomyces cerevisiae. J Biol Chem 286: 25027-25038.

3. Hu J, Shibata Y, Zhu PP, Voss C, Rismanchi N, et al. (2009) A class of dynamin-like GTPases involved in the generation of the tubular ER network. Cell 138: 549-561.

4. Anwar K, Klemm RW, Condon A, Severin KN, Zhang M, et al. (2012) The dynamin-like GTPase Sey1p mediates homotypic ER fusion in S. cerevisiae. J Cell Biol 197: 209-217.

5. Chen S, Novick P, Ferro-Novick S (2012) ER network formation requires a balance of the dynamin-like GTPase Sey1p and the Lunapark family member Lnp1p. Nat Cell Biol 14: 707-716.

6. Janke C, Magiera MM, Rathfelder N, Taxis C, Reber S, et al. (2004) A versatile toolbox for PCR-based tagging of yeast genes: new fluorescent proteins, more markers and promoter substitution cassettes. Yeast 21: 947-962.

7. Xu K, Huang TS, Nagy PD (2012) Authentic in vitro replication of two tombusviruses in isolated mitochondrial and endoplasmic reticulum membranes. J Virol 86: 12779-12794.

8. Barajas D, Li Z, Nagy PD (2009) The Nedd4-type Rsp5p ubiquitin ligase inhibits tombusvirus replication by regulating degradation of the p92 replication protein and decreasing the activity of the tombusvirus replicase. J Virol 83: 11751-11764.

9. Wang RY, Stork J, Nagy PD (2009) A key role for heat shock protein 70 in the localization and insertion of tombusvirus replication proteins to intracellular membranes. J Virol 83: 3276-3287.

10. Patel SK, Indig FE, Olivieri N, Levine ND, Latterich M (1998) Organelle membrane fusion: a novel function for the syntaxin homolog Ufe1p in ER membrane fusion. Cell 92: 611-620.

11. Busquets A, Keim V, Closa M, del Arco A, Boronat A, et al. (2008) Arabidopsis thaliana contains a single gene encoding squalene synthase. Plant Mol Biol 67: 25-36.

12. Barajas D, Xu K, de Castro Martin IF, Sasvari Z, Brandizzi F, et al. (2014) Co-opted Oxysterol-Binding ORP and VAP Proteins Channel Sterols to RNA Virus Replication Sites via Membrane Contact Sites. PLoS Pathog 10: e1004388.

13. Xu K, Lin JY, Nagy PD (2014) The hop-like stress-induced protein 1 cochaperone is a novel cell-intrinsic restriction factor for mitochondrial tombusvirus replication. J Virol 88: 9361-9378.

14. Xu K, Nagy PD (2015) RNA virus replication depends on enrichment of phosphatidylethanolamine at replication sites in subcellular membranes. Proc Natl Acad Sci U S A 112: E1782-1791.

15. Li Z, Barajas D, Panavas T, Herbst DA, Nagy PD (2008) Cdc34p ubiquitin-conjugating enzyme is a component of the tombusvirus replicase complex and ubiquitinates p33 replication protein. J Virol 82: 6911-6926.

16. Rajendran KS, Pogany J, Nagy PD (2002) Comparison of turnip crinkle virus RNA-dependent RNA polymerase preparations expressed in Escherichia coli or derived from infected plants. J Virol 76: 1707-1717.

17. Pogany J, Nagy PD (2008) Authentic replication and recombination of Tomato bushy stunt virus RNA in a cell-free extract from yeast. J Virol 82: 5967-5980.

18. Xu K, Nagy PD (2016) Enrichment of Phosphatidylethanolamine in Viral Replication Compartments via Co-opting the Endosomal Rab5 Small GTPase by a Positive-Strand RNA Virus. PLoS Biol 14: e2000128.

19. Xu K, Nagy PD (2015) RNA virus replication depends on enrichment of phosphatidylethanolamine at replication sites in subcellular membranes. Proc Natl Acad Sci U S A 112: E1782-E1791.

20. Nawaz-Ul-Rehman MS, Prasanth KR, Xu K, Sasvari Z, Kovalev N, et al. (2016) Viral Replication Protein Inhibits Cellular Cofilin Actin Depolymerization Factor to Regulate the Actin Network and Promote Viral Replicase Assembly. PLoS Pathog 12: e1005440.

21. Panavas T, Nagy PD (2003) Yeast as a model host to study replication and recombination of defective interfering RNA of Tomato bushy stunt virus. Virology 314: 315-325.

22. Ren Y, Yip CK, Tripathi A, Huie D, Jeffrey PD, et al. (2009) A structure-based mechanism for vesicle capture by the multisubunit tethering complex Dsl1. Cell 139: 1119-1129.

23. Tripathi A, Ren Y, Jeffrey PD, Hughson FM (2009) Structural characterization of Tip20p and Dsl1p, subunits of the Dsl1p vesicle tethering complex. Nat Struct Mol Biol 16: 114-123.

24. Barajas D, Kovalev N, Qin J, Nagy PD (2015) Novel Mechanism of Regulation of Tomato Bushy Stunt Virus Replication by Cellular WW-Domain Proteins. J Virol 89: 2064-2079.

25. Panaviene Z, Panavas T, Serva S, Nagy PD (2004) Purification of the cucumber necrosis virus replicase from yeast cells: role of coexpressed viral RNA in stimulation of replicase activity. J Virol 78: 8254-8263.

26. Kovalev N, Nagy PD (2013) Cyclophilin a binds to the viral RNA and replication proteins, resulting in inhibition of tombusviral replicase assembly. J Virol 87: 13330-13342.

27. Kovalev N, Pogany J, Nagy PD (2012) A Co-Opted DEAD-Box RNA Helicase Enhances Tombusvirus Plus-Strand Synthesis. PLoS Pathog 8: e1002537.

28. Schmitt HD (2010) Dsl1p/Zw10: common mechanisms behind tethering vesicles and microtubules. Trends Cell Biol 20: 257-268.

29. Tagaya M, Arasaki K, Inoue H, Kimura H (2014) Moonlighting functions of the NRZ (mammalian Dsl1) complex. Front Cell Dev Biol 2: 25.

30. Vukasinovic N, Zarsky V (2016) Tethering Complexes in the Arabidopsis Endomembrane System. Front Cell Dev Biol 4: 46.
