# Supplementary material for: Assembly-hub function of ER-localized SNARE proteins in biogenesis of tombusvirus replication compartment
Source: PLoS Pathog. 2018 May 10;14(5):e1007028. doi: 10.1371/journal.ppat.1007028 (PMC5963807; doi:10.1371/journal.ppat.1007028)
Supplement: S1 Table — (DOCX) [file ppat.1007028.s006.docx]

**S1 Table. The names and sequences of oligo primers used in this study**

| Name | Sequence (5’-to-3’) |
| --- | --- |
| #1295 | CGGCGGATCCCTTGTACAGCTCGTCCATGCCGA |
| #1858 | GGCGGGATCCATGAAGATGTTAACTAAATTTG |
| #1859 | GGCGCTCGAGTTATACACGTATTCTTAATCCGGA |
| #1941 | CGGCAGATCTGTGAGCAAGGGCGAGGAGCTGTTCA |
| #2632 | GGAGGGATCCAAAGATGGAGGGTTTGAAGGCTG |
| #2859 | TAATACGACTCACTATAGGAACCAAATCATTCATGTTGCTCTC |
| #2860 | TAGTGTATGTGATATCCCACCAA |
| #3132 | GCCGGATCCATGGGTTTGTTTGCCTCTAAGTTGTTC |
| #3665 | CGCGCTCGAGCTATTTGACACCGAGGGATTC |
| #5220 | CCAGAGATCTATGGTCGTGACATTTTTGC |
| #5221 | CCAGGCTAGCTCATCATGTGAGACGATCC |
| #5224 | CCAGAGATCTATGGAGTCTCTTTTTCCTAACAAAGG |
| #5225 | CCAGGCTAGCATCATCTAGAGCAGTGCCTCTAATTTC |
| #5298 | CGCCAGATCTATGGGAATCGGCAAAACG |
| #5299 | CGCCGCTAGCTGTGACTCGGATCAACATGACA |
| #5362 | CGCCGGATCCATGATGTCTGATTTAACACCAATATTCC |
| #5364 | CGCCGGATCCATGGCTGAAACTTCCAACG |
| #5365 | CGCCGCTAGCTTATAGGGCCGGGAATAAT |
| #5398 | GCGCTCGAGAGCAGTTCTTCCAGCAGCTC |
| #5400 | CGCCGGATCCACATTCAAAGGGATGGCTTG |
| #5401 | CGCGCTCGAGGTTCAACGCAGACATTTCCA |
| #5402 | CGCCGGATCCATGTCAAAAATTAGAGACAGAACAGAA |
| #5403 | CGCGCTCGAGTCTGTTGTACGGTGCAAATCA |
| #5483 | ATGTCGAAAGCTACATATAAGGAACGTGCTGCTACTCATCCTACGTACGCTGCAGGTCGA |
| #5484 | TTAGTTTTGCTGGCCGCATCTTCTCAAATATGCTTCCCAGCCATCGATGAATTCGAGCTC |
| #5533 | CGCCGTCGACGGTTCTGCTGCTGCTGTTTC |
| #5588 | CGCGGATCCTTGGACTTGCATTTGAGTATCG |
| #5589 | CGCCTCGAGTTTCTCAAGTATGAAAATCACCTG |
| #5590 | CGCCTCGAGTTAACCTACATAATCTAGGAACAATATAAAAAC |
| #5648 | TGTCCAGGAGATCTGCTAGCTT |
| #5742 | GCCCTGCAGAGTTGAGTTTTTCAAACTGTTACTTAACCATTC |
| #5774 | CGCACTAGTATGATGTCTGATTTAACACCAATATTCC |
| #5775 | GCGCCATGGTTAACCTACATAATCTAGGAACAATATAAAAACA |
| #5871 | CGCGGATCCATGGGAAAGCTATTACAATTGGCATT |
| #5872 | CGCCTCGAGTCACGCTCTGTGTAAAGTGTATATATAATAAA |
| #5880 | CGCGGATCCATGTCGAGATTCAGAGACAGGA |
| #5881 | CGCCTCGAGTTAACTGTACCAATCCAAGAACAA |
| #5905 | GGAAGATCTATGGTGAGCAAGGGCGAG |
| #5908 | GGAAGATCTATGGGCAGCGTGCAGCTC |
| #5971 | CGCGCTAGCCGCTCTGTGTAAAGTGTATATATAATAAA |
| #6055 | CGCCCTCGAGCTATGTGACTCGGATCAACATGA |
| #6056 | GGCGGATCCTTGTACAGCTCGTCCATGCC |
| #6069 | GCGCGGATCCGTCCTCGATGTTGTGGC |
| #6076 | CGCGGATCCCATCATCATCATCATCATATGGGGAGCTTGGGGACG |
| #6077 | CGGCTCGAGTCAACTAGTGTTTGCTCTGAGATATGCAAAG |
| #6097 | CGCCTCGAGTTATAGGGCCGGGAATAAT |
